# Prospective cohort study evaluating the association between influenza vaccination and neurodegenerative diseases

**DOI:** 10.1038/s41541-024-00841-z

**Published:** 2024-03-02

**Authors:** Houyu Zhao, Xuan Zhou, Kexin Fu, Yunxiao Duan, Qiaorui Wen, Shengfeng Wang, Siyan Zhan

**Affiliations:** 1 School of Medicine, Chongqing University, Chongqing, China; 2 Department of Epidemiology and Biostatistics, School of Public Health, Peking University, Beijing, China; 3 Key Laboratory of Epidemiology of Major Diseases (Peking University), Ministry of Education, Beijing, China; 4 Department of Biostatistics, School of Global Public Health, New York University, New York, USA; 5 Department of Biostatistics, Yale School of Public Health, Yale University, New Haven, USA; 6 Research Center of Clinical Epidemiology, Peking University Third Hospital, Beijing, China; 7 Center for Intelligent Public Health, Institute for Artificial Intelligence, Peking University, Beijing, China

**Keywords:** Alzheimer's disease, Parkinson's disease, Epidemiology

## Abstract

The effect of influenza vaccination (FluVac) on the risk of neurodegenerative diseases has not been well evaluated in prospective populations. We aimed to assess the association between FluVac and the risk of dementia and Parkinson’s disease (PD) in people aged 60 years or older through a prospective population-based cohort from the UK Biobank. A time-varying Cox regression model adjusted for baseline and repeatedly measured covariates was used to estimate the hazard ratio (HR) and 95% confidence interval (CI) of the association between influenza vaccination and risk of dementia/PD. We took into account 70,938 participants in the cohort, including 38,328 participants who got vaccinated. During a median follow-up period of 12.2 years, 2087 incident dementia cases occurred, including 281 cases who received FluVac and 1806 cases who were not vaccinated. In addition, 742 incident PD cases occurred, among whom 131 cases received FluVac and 611 PD cases did not receive FluVac. FluVac was associated with reduced dementia risk with an HR of 0.83 (95% CI, 0.72–0.95) but was not associated with PD incidence (HR = 1.07; 95% CI, 0.87–1.32) after controlling baseline and repeatedly measured covariates. Further, among all dementia cases, there were 733 Alzheimer’s disease (AD) (94 vaccinated cases and 639 non-vaccinated cases), 307 vascular dementia (VD) (34 vaccinated cases and 273 non-vaccinated cases), and 1047 cases with other dementias (OD) (153 vaccinated cases and 894 non-vaccinated cases). The HRs for the associations between FluVac and AD, VD, and OD were 0.79 (95% CI, 0.63–1.00), 0.58 (95% CI, 0.39–0.86), and 0.94 (95% CI, 0.78–1.14) respectively. A dose-response relationship was found in the association between FluVac and dementia but not in the association with PD. A major limitation of the study is the low accuracy in the diagnosis of dementia subtypes, namely AD, VD, and OD. However, Results of sensitivity analyses were consistent with the primary analyses. In conclusion, influenza vaccination is significantly associated with a reduced risk of incident dementia but not PD in community-dwelling adults in the UK Biobank population.

## Introduction

Dementia and Parkinson’s disease (PD) are the most common neurodegenerative diseases, which result in irreversible decline in cognitive and motor functioning and affect millions of people worldwide. It is estimated that there are more than 55 million dementia patients and over six million people living with PD worldwide^1,2^. As populations are growing and aging, prevalence of these diseases is predicted to increase substantially over the next decades^2–4^. However, to date, there are no definitive treatments for dementia and PD that can modify the pathological process of these disorders. An alternative strategy that repositions and repurposes available interventions for other conditions to prevent or treat dementia and PD has attracted accumulating attention in recent years.

Previous studies indicated that infections can increase the risk of dementia and PD in older populations^5,6^, thus, vaccinations may play a promising role in decreasing the risk of these diseases. Influenza vaccination (FluVac) is the key public health intervention to reduce the morbidity and mortality of influenza, with the traditional strategy to vaccinate those at high risk of severe complications, such as the elderly, pregnant women, children, and adults with underlying chronic medical conditions^7^. In recent years, several epidemiological studies found a protective effect of FluVac against dementia in older adults based mainly on retrospective claim data or electronic health records^8,9^, thus suffering from limitations of real-world data studies, such as unmeasured confounders of lifestyle factors^10^. However, the population in these studies were only from North America and the West Pacific, thus the results may not be directly extrapolated to other populations. Further, some of current studies were based on prevalent receiver of FluVac, which could induce selection bias^11^. Moreover, no population-based study has investigated the association between FluVac and the incidence of PD. Given this background, in this study we assessed the associations between FluVac and the incidence of dementia and PD through a prospective cohort of community-dwelling adults based on the UK Biobank (UKB). Annual vaccination against influenza is well established in the UK and much of the influenza vaccine program is delivered through primary care^7^, of which the data is available in the UKB through external data link^12^.

## Results

### Basic characteristics

A total of 70,938 participants were included in the final analyses (Supplementary Fig. 1). In the first year 4443 participants got vaccinated and the cumulative number of participants receiving FluVac increased obviously in the first seven years of follow-up (Supplementary Fig. 2). Overall, 38,328 participants got vaccinated in the study period and the Table 1 shows the baseline characteristics of participants who were vaccinated and those not vaccinated. The mean age was 64.4 (3.1) years and 46.4% (32,884) participants were male. The two groups of participants were significantly different in most of the baseline characteristics except for family history of dementia and PD, smoking status, healthy diet, regular physical activity, and mental health score. Vaccinated participants tended to have higher Charlson comorbidity index (CCI) and were more likely to receive drugs for chronic diseases.

**Table 1:** Baseline characteristics of participants in the study cohort based on the UK Biobank

	Overall	Ever vaccinated	Non vaccinated	SMD
Male, *n* (%)	32,884 (46.4)	18,071 (47.1)	14,813 (45.4)	0.035
Age, mean (std)	64.4 (3.1)	64.0 (2.7)	64.9 (3.5)	0.294
Age group, *n* (%)				
60–64	43,802 (61.7)	25,642 (66.9)	18,160 (55.7)	0.232
≥65	27,136 (38.3)	12,686 (33.1)	14,450 (44.3)	0.232
ApoE 4 gene type				
ε4 noncarrier	49,656 (70.0)	26,915 (70.2)	22,741 (69.7)	0.011
ε4 carrier	19,531 (27.5)	10,537 (27.5)	8994 (27.6)	0.002
Unknown	1751 (2.5)	876 (2.3)	875 (2.7)	0.026
Family history of dementia, *n* (%)	10,922 (15.4)	5857 (15.3)	5065 (15.5)	0.007
Family history of PD, *n* (%)	3392 (4.8)	1821 (4.8)	1571 (4.8)	0.003
Education, *n* (%)				
University/college degree	18,634 (26.3)	10,170 (26.5)	8464 (26.0)	0.013
A levels/AS levels or equivalent	6205 (8.7)	3310 (8.6)	2895 (8.9)	0.009
O-levels/GCEs/CSEs or equivalent	16,419 (23.1)	8732 (22.8)	7687 (23.6)	0.019
NVQ/HND/HNC/other professional qualification	10,176 (14.3)	5460 (14.2)	4716 (14.5)	0.006
Others	18,516 (26.1)	10,138 (26.5)	8378 (25.7)	0.017
Unknown	988 (1.4)	518 (1.4)	470 (1.4)	0.008
TDI, mean (std)	−1.6 (2.9)	−1.6 (2.9)	−1.6 (2.9)	0.022
TDI, *n* (%)				
≤−3.64	19,133 (27.0)	10,334 (27.0)	8799 (27.0)	<0.001
≤−2.14	19,045 (26.8)	10,380 (27.1)	8665 (26.6)	0.012
≤0.55	17,766 (25.0)	9736 (25.4)	8030 (24.6)	0.018
>0.55	14,916 (21.0)	7833 (20.4)	7083 (21.7)	0.031
Unknown	78 (0.1)	45 (0.1)	33 (0.1)	0.005
Average total household income, *n* (%)				
<18,000	19,739 (27.8)	10,382 (27.1)	9357 (28.7)	0.036
18,000–30,999	19,023 (26.8)	10,434 (27.2)	8589 (26.3)	0.020
31,000–51,999	12,176 (17.2)	6717 (17.5)	5459 (16.7)	0.021
>52,000	7321 (10.3)	4013 (10.5)	3308 (10.1)	0.011
Unknown	12,679 (17.9)	6782 (17.7)	5897 (18.1)	0.010
Study center region, *n* (%)				
England	62,154 (87.6)	31,432 (82.0)	30,722 (94.2)	0.384
Wales	3492 (4.9)	2600 (6.8)	892 (2.7)	0.191
Scotland	5292 (7.5)	4296 (11.2)	996 (3.1)	0.321
BMI, mean (std)	27.4 (4.7)	27.5 (4.6)	27.4 (4.7)	0.019
BMI, *n* (%)				
<25	21,103 (29.7)	11,196 (29.2)	9907 (30.4)	0.026
<18.5	290 (0.4)	145 (0.4)	145 (0.4)	0.010
<30	32,005 (45.1)	17,555 (45.8)	14,450 (44.3)	0.030
≥30	17,208 (24.3)	9275 (24.2)	7933 (24.3)	0.003
Unknown	332 (0.5)	157 (0.4)	175 (0.5)	0.019
Smoking status, *n* (%)				
Never	35,742 (50.4)	19,204 (50.1)	16,538 (50.7)	0.012
Previous smoking	28,877 (40.7)	15,754 (41.1)	13123 (40.2)	0.018
Current smoking	5918 (8.3)	3162 (8.2)	2756 (8.5)	0.007
Unknown	401 (0.6)	208 (0.5)	193 (0.6)	0.007
Drinking status, *n* (%)				
Never	3248 (4.6)	1627 (4.2)	1621 (5.0)	0.035
Previous drinking	2540 (3.6)	1298 (3.4)	1242 (3.8)	0.023
Current drinking	64,989 (91.6)	35,323 (92.2)	29,666 (91.0)	0.043
Unknown	161 (0.2)	80 (0.2)	81 (0.2)	0.008
Health diet, *n* (%)	21,949 (30.9)	11,822 (30.8)	10,127 (31.1)	0.005
Tea intake (/day), *n* (%)				
0 cups	11,301 (15.9)	5979 (15.6)	5322 (16.3)	0.020
1–2 cup	15,433 (21.8)	8454 (22.1)	6979 (21.4)	0.016
3–4 cups	22,028 (31.1)	12,024 (31.4)	10,004 (30.7)	0.015
≥5 cups	21,921 (30.9)	11,739 (30.6)	10,182 (31.2)	0.013
Unknown	255 (0.4)	132 (0.3)	123 (0.4)	0.005
Coffee intake (/day), *n* (%)				
0 cups	18,416 (26.0)	9787 (25.5)	8629 (26.5)	0.021
1 cup	15,310 (21.6)	8266 (21.6)	7044 (21.6)	0.001
2 cups	14,226 (20.1)	7759 (20.2)	6467 (19.8)	0.010
≥3 cups	22,756 (32.1)	12,399 (32.3)	10,357 (31.8)	0.013
Unknown	230 (0.3)	117 (0.3)	113 (0.3)	0.007
Regular physic activity, *n* (%)	56,456 (79.6)	30,462 (79.5)	25,994 (79.7)	0.006
Self-health rating				
Excellent	11,462 (16.2)	6208 (16.2)	5254 (16.1)	0.002
Good	42,885 (60.5)	23,429 (61.1)	19,456 (59.7)	0.030
Fair	13,839 (19.5)	7374 (19.2)	6465 (19.8)	0.015
Poor	2374 (3.3)	1130 (2.9)	1244 (3.8)	0.048
Unknown	378 (0.5)	187 (0.5)	191 (0.6)	0.013
Mental health score, *n* (%)				
≤2	26,578 (37.5)	14,330 (37.4)	12,248 (37.6)	0.004
≤4	15,758 (22.2)	8514 (22.2)	7244 (22.2)	<0.001
≤7	16,607 (23.4)	9012 (23.5)	7595 (23.3)	0.005
≥8	10,996 (15.5)	5911 (15.4)	5085 (15.6)	0.005
Unknown	999 (1.4)	561 (1.5)	438 (1.3)	0.010
Social isolation, *n* (%)				
Least isolated	32,985 (46.5)	18,196 (47.5)	14,789 (45.4)	0.043
Moderately isolated	27,601 (38.9)	14,786 (38.6)	12,815 (39.3)	0.015
Most isolated	9146 (12.9)	4750 (12.4)	4396 (13.5)	0.032
Unknown	1206 (1.7)	596 (1.6)	610 (1.9)	0.024
Depression, *n* (%)	12,186 (17.2)	6473 (16.9)	5713 (17.5)	0.017
Charlson comorbidity index, *n* (%)				
0	48,836 (68.8)	26,721 (69.7)	22,115 (67.8)	0.041
1	11,189 (15.8)	5935 (15.5)	5254 (16.1)	0.017
2	7965 (11.2)	4231 (11.0)	3734 (11.5)	0.013
>2	2948 (4.2)	1441 (3.8)	1507 (4.6)	0.043
Influenza vaccination invitation, *n* (%)	2339 (3.3)	1207 (3.1)	1132 (3.5)	0.018
Medication use, *n* (%)				
Aspirin	6703 (9.4)	4052 (10.6)	2651 (8.1)	0.084
Angiotensin-converting enzyme inhibitors	6495 (9.2)	3898 (10.2)	2597 (8.0)	0.077
Angiotensin receptor blockers	2796 (3.9)	1686 (4.4)	1110 (3.4)	0.051
Glucose lowering agents	1486 (2.1)	899 (2.3)	587 (1.8)	0.038
Statins	11316 (16.0)	6938 (18.1)	4378 (13.4)	0.129
Calcium channel blockers	5398 (7.6)	3283 (8.6)	2115 (6.5)	0.079
Beta blocking agents	5666 (8.0)	3495 (9.1)	2171 (6.7)	0.091
Diuretics	6763 (9.5)	4172 (10.9)	2591 (7.9)	0.101
Proton-pump inhibitors	8225 (11.6)	5050 (13.2)	3175 (9.7)	0.108

*ApoE* apolipoprotein E, *TDI* Townsend deprivation index, *BMI* body mass index, *SMD* standardized mean difference.

### Association between FluVac and risk of dementia/PD

During a median follow-up period of 12.2 years (interquartile range, 11.4–13.0) and 817,936 person-years, 2087 incident dementia cases occurred, including 281 dementia cases in subjects who received FluVac and 1806 dementia cases in subjects who did not receive FluVac. Moreover, 742 incident PD cases occurred, including 131 PD cases in participants who received FluVac and 611 PD cases in participants who did not receive FluVac. FluVac was associated with dementia risk with an HR of 0.74 (95% CI, 0.65–0.84) but not PD incidence with an HR of 0.98 (95% CI, 0.80–1.20) in the crude analyses controlling no potential confounders (Table 2). After controlling all baseline and repeatedly measured covariates in the time-varying model, FluVac was associated with a 17% reduction in the risk of dementia (HR = 0.83, 95% CI, 0.72–0.95) but not associated with the PD incidence (HR = 1.07, 95% CI, 0.87–1.32). Further, among incident dementia cases, there were 733 Alzheimer’s disease (AD) (94 in vaccinated cases and 639 in non-vaccinated cases), 307 vascular dementia (VD) (34 in vaccinated cases and 273 in non-vaccinated cases), and 1047 cases with other dementias (OD) (153 in vaccinated cases and 894 in non-vaccinated cases). The HRs for the association between FluVac and AD, VD, and OD were 0.79 (95% CI, 0.63–1.00), 0.58 (95% CI, 0.39–0.86), and 0.94 (95% CI, 0.78–1.14) respectively.

**Table 2:** Association between influenza vaccination and risk of dementia or Parkinson’s disease, HR (95% CI)

Outcomes	Crude model	Adjust for sex and age	Adjust for baseline covariates^a^	Adjust for baseline and time-varying covariates^b^
Primary endpoints				
All-cause dementia	0.74 (0.65–0.84)	0.82 (0.72–0.94)	0.85 (0.74–0.97)	0.83 (0.72–0.95)
Parkinson’s disease	0.98 (0.80–1.20)	1.03 (0.84–1.26)	1.08 (0.88–1.32)	1.07 (0.87–1.32)
Secondary endpoints				
Alzheimer’s disease	0.72 (0.57–0.90)	0.81 (0.64–1.01)	0.79 (0.63–1.00)	0.79 (0.63–1.00)
Vascular dementia	0.58 (0.40–0.84)	0.68 (0.46–1.00)	0.62 (0.42–0.93)	0.58 (0.39–0.86)
Other dementia	0.80 (0.67–0.96)	0.88 (0.73–1.05)	0.97 (0.81–1.17)	0.94 (0.78–1.14)

^a^Adjusted for baseline values of the all covariates in Table 1.

^b^Adjusted for all covariates in Table 1. Time-varying factors were measured in a one-year interval lag before every influenza season, including influenza vaccination invitation, comorbidities and medication use (aspirin, glucose-lowering agents, statins, calcium channel blockers, angiotensin-converting enzyme inhibitors, angiotensin receptor blockers, proton-pump inhibitors, beta blocking agents, and diuretics).

### Subgroup analyses and cumulative influenza vaccination

**Fig. 1:**
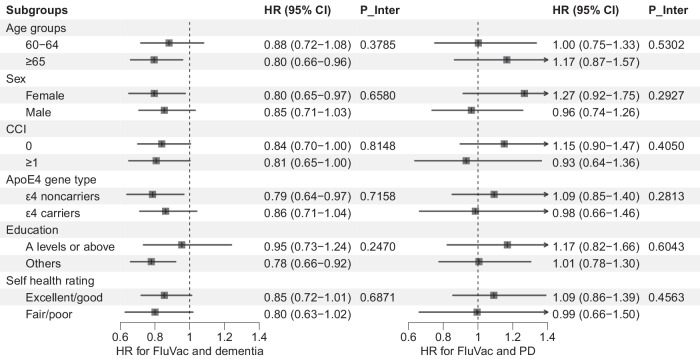
Association between influenza vaccination and risk of dementia or Parkinson’s disease in different subpopulations. *FluVac influenza vaccination; PD Parkinson’s disease; CCI Charlson comorbidity index; P_Inter *P* value of interaction. Error bars represent 95% confidence intervals.

**Fig. 2:**
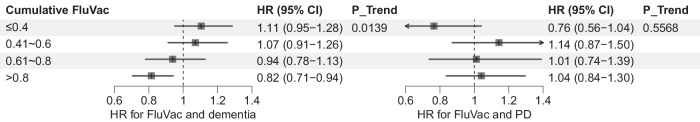
Dose-response relationship of the association between influenza vaccination and risk of dementia or Parkinson’s disease in the UK Biobank. *FluVac influenza vaccination; PD Parkinson’s disease; P_Trend *P* value of trend. Error bars represent 95% confidence intervals.

In the subgroup analyses (Fig. 1), we did not find any significant interactions between FluVac and baseline characteristics in the associations between FluVac and dementia/PD (p for interactions >0.05 for all subgroup analyses). For instance, the HRs were 0.79 (95% CI, 0.64–0.97) and 0.86 (95% CI, 0.71–1.04) in ApoE ε4 noncarriers and carriers for the risk of dementia (p for interaction 0.7158); in participants with education of A level or above and other education level the HRs were 0.95 (95% CI, 0.73–1.24) and 0.78 (95% CI, 0.66–0.92) with an insignificant interaction (*p* 0.2470); and the HRs were 1.00 (95% CI, 0.75–1.33) and 1.17 (95% CI, 0.87–1.57) in participants aged <65 years and ≥65 years respectively for PD risk (*p* for interaction 0.5302). Moreover, we found a significant dose-response relationship in the association between FluVac and dementia incidence (Fig. 2), the fully adjusted HRs for the average number of influenza vaccinations per year (AvgFluVac) ≤ 0.4, 0.41 ~ 0.6, 0.61 ~ 0.8, and >0.8 were 1.11 (95% CI, 0.95–1.28), 1.07 (95% CI, 0.91–1.26), 0.94 (95% CI, 0.78–1.13), and 0.82 (95% CI, 0.71–0.94) respectively (*p* for trend 0.0139). However, there was no dose-response association between FluVac and PD (*p* for trend 0.5568). Results of continuous AvgFluVac are presented in Supplementary Fig. 3 which were in line with the results modeling the AvgFluVac as categorical variable and indicated that participants with an AvgFluVac larger than 0.5 had a significantly lower risk of dementia but not PD.

### Sensitivity analyses

**Fig. 3:**
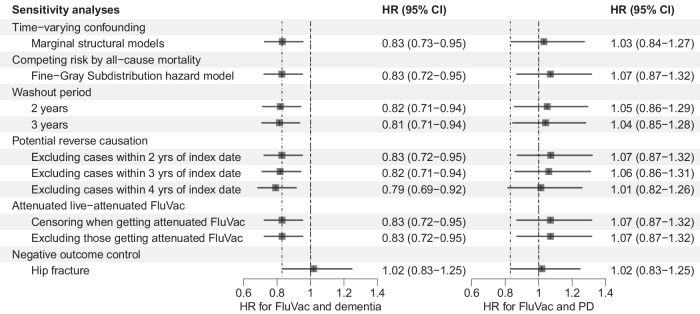
Results of sensitivity analyses. *FluVac influenza vaccination; PD Parkinson’s disease. Error bars represent 95% confidence intervals.

Sensitivity analyses were generally consistent with the primary analyses (Fig. 3). First, in the marginal structural model (MSM) HRs were 0.83 (95% CI, 0.73–0.95) and 1.03 (95% CI, 0.84–1.27) for dementia and PD risk respectively after adjusting confounding and censoring using inverse probability of treatment weighting (IPTW) and censoring weighting (IPCW). Second, a total of 6120 deaths (5194 never vaccinated and 926 ever vaccinated) occurred during following-up. However all-cause mortality did not have impacts on the results and the competing risk model got highly consistent estimates, with an HR of 0.83 (95% CI, 0.72–0.95) for dementia risk and 1.07 (95% CI, 0.87–1.32) for PD risk. Third, alternative washout periods of two years got HRs of 0.82 (95% CI, 0.71–0.94) and 1.05 (95% CI, 0.86–1.29) for the risk of dementia and PD. Further analyses with a three-year washout period were also in line with the primary analyses. Fourth, when excluding cases within two, three, and four years of follow-up, the HRs for dementia were 0.83 (95% CI, 0.72–0.95), 0.82 (95% CI, 0.71–0.94), and 0.79 (95% CI, 0.69–0.92) respectively and all HRs were not significant for PD risk. Fifth, there were only 16 participants received live-attenuated influenza vaccine and the sensitivity analyses excluding or censoring these participants had little impact on the results. Finally, we found no association between FluVac and hip fracture, with an HR of 1.02 (95% CI, 0.83–1.25).

## Discussion

In a prospective cohort based on the UKB our analyses indicated that FluVac was associated with a reduced risk of incident dementia but not PD during a median follow-up of over 12 years. A dose-response relationship was also found in the association between FluVac and dementia but not PD. Various subgroup analyses and sensitivity analyses were generally consistent with the primary analyses and the negative control outcome (NCO) analysis found no association between FluVac and hip fracture, showing the robustness of our results against potential unmeasured confounding.

Our results aligned closely with several retrospective cohort studies, all of which reported a negative association between FluVac and dementia with HRs ranging from 0.60–0.86^13–17^. The discrepancy in the association magnitude might be attributed to heterogeneity in population, methodology, and outcome definition. For instance, the studies conducted by Luo et al.^15^, Lee et al.^16^, Liu et al.^17^, and Wiemken et al.^14^ defined new receivers of FluVac with only six months of washout period and measured FluVac exposure in the follow-up period. However, they did not explicitly show that FluVac status was modeled as a time-dependent variable, which could correctly divide the follow-up time into non-exposed before receiving FluVac. Consequently, these studies might have a risk of immortal-time bias, which could overestimate the potentially protective effect of FluVac^18^. Moreover, all of these studies did not consider the latency time of dementia except the one conducted by Wiemken et al.^14^, which required a minimum follow-up of 90 days and excluded cases that occurred in this period, thus latency time bias and reverse causality could not be ruled out in these studies^19^. In contrast, we minimized the above bias through a longer washout period, time-vary exposure, and longer latency time, as well as a series of sensitivity analyses against potential bias. Furthermore, the dose-response relationship in the association between FluVac and dementia, which indicated a potential risk reduction with an increasing number of vaccinations, corroborated the findings proposed by Luo et al.^15^ and Liu et al.^17^. In addition, similar views were presented by Wiemken et al.^14^, suggesting that patients with 1, 2, or 3–5 vaccines versus none exhibited similar risks for dementia but patients with 6 or more influenza vaccines had a significantly lower risk for dementia. Despite these studies, our results additionally illustrated that receiving FluVac continuously was also important for the potential protective effects against dementia. However, the absence of associations in most AvgFluVac categories indicated that our results need further repetition in other populations. Moreover, our study identified associations of FluVac with the risk of AD and VD, which were consistent with previous studies by Verreault et al.^20^, Luo et al.^15^, and Bukhbinder et al.^13^.

However, studies investigating the association between FluVac and PD incidence were very scarce and our analyses did not reveal a significant association between FluVac and the risk of incident PD. The association between influenza infection and PD is also controversial. A meta-analysis combining data from four small, case-control studies indicated a lack of a significant association between influenza infection and PD^21^. However, a recent large-scale case-control study with more than 10 thousand PD cases suggested that influenza was associated with diagnoses of PD more than 10 years after infection^22^. One hypothesis was proposed to explain these contradictory results, that is that the role of influenza on PD risk may be specific to the circulating virus strain^6,22^. Therefore, studies with more details of FluVac compositions and longer follow-up may provide additional valuable evidence on the association between FluVac and PD risk.

Influenza vaccination might have a protective effect on dementia by preventing infections, which in recent studies are illustrated to increase risk of dementia^5^. However, so far, research on the mechanism of influenza vaccines in affecting dementia risk has been mainly conducted on animals^8^ and several potential mechanisms directly linking influenza vaccination to dementia have been proposed. The first is non-influenza-specific training of the immune system. Given that human studies have not identified evidence that influenza leads to a risk of dementia^8^, it suggests that the association between vaccination and dementia might not be entirely explained by influenza prevention, and there might be other multi-effects at play^9^. Some studies have shown that vaccination might lower the risk of dementia by training the immune system to strengthen immunity against pathogens^23^, reducing central nervous system inflammation and subsequent off-target effects caused by the progression of dementia. This offers non-specific protection against neurotoxic inflammation and oxidative stress related to infectious diseases, which might significantly reduce cerebral vascular damage. This could perhaps explain the inverse association between VD and vaccination found in studies^9,14^. Moreover, FluVac might also increase the activity of microglial cells, leading to the clearance of β-amyloid proteins, disrupting the Treg-regulated immune system, and exerting immunomodulatory effects on amyloid precursor protein (APP)/presenilin 1^8,9^, thus contribute to some protective effects on AD. In addition to this, other potential mechanisms include non-influenza-specific changes in adaptive immunity mediated by lymphocyte cross-reaction and influenza-specific mechanisms, including mitigating secondary damage from influenza infections and/or epitope similarity between influenza proteins and AD pathology^13^.

Although a series of observational studies, including ours, have indicated an association between influenza vaccination and dementia, the real mechanism by which the influenza vaccine provides potential protection against dementia, with the anti-inflammatory mechanism potentially being one of them, still necessitates further clinical trials and biochemical research to offer conclusive evidence^13,16^. This is necessary to explain and support the results of epidemiological studies and the implementation of corresponding public health measures.

The influenza vaccine, as a potential public health intervention to prevent and reduce the risk of dementia, is relatively inexpensive, low-risk, and easily accessible^8,14^, and can further reduce societal burdens and caregiving costs^17^. More importantly, the vaccination coverage for influenza is low among the elderly population prone to dementia^9^. These facts emphasize the need to increase influenza vaccination among the elderly. This approach might be more effective than most other preventive measures, like public policies on dementia prevention that involve changing the general population’s lifestyles and dietary habits, which are difficult to implement^14^. Therefore, cost-effectiveness and policy promoting influenza vaccination may consider not only the short-term effects of influenza vaccine in preventing influenza but also some long-term health benefits including reduced risk of dementia.

To our knowledge, this study is the first prospective cohort analysis to simultaneously assess the associations between FluVac and the risk of dementia and PD, which are the most common neurodegenerative disorders and share some pathological mechanisms, such as oxidative stress and mitochondrial dysfunction^24^. We applied a new-user design and evaluated the potential effects of time-varying FluVac exposure with controlling for time-invariant and time-varying confounders, which were rarely considered in previous studies. This design allowed us to accurately measure exposure in fine detail and control for possible selection bias, such as depletion of susceptibles in study based on prevalent users^11^. However, our study has limitations. Our results should not be interpreted as causal effects on account of the observational nature and more studies in other populations are needed to repeat this study. Since secondary outcomes related to a dementia syndrome (AD, VD, and OD) were identified with low diagnostic accuracy^25^, the occurrence of such outcomes was susceptible to misclassification bias. Accordingly, a quite high percentage (about 50.2%) of the 2087 cases with incident dementia were diagnosed as cases with OD, namely with a dementia syndrome not due to AD or VD. Given such low accuracy in the diagnosis of disorders associated with dementia, further studies on the association between FluVac and dementia subtypes are needed. In addition, because most of the vaccination records in the primary care data contained no detailed information on the compositions of influenza vaccine, we did not conduct an in-depth analysis of the potential effects of different FluVac compositions which were usually annually updated. Future studies focusing on specific effects of different FluVac compositions, such as valence and viral strain, could provide more valuable insights into the mechanism of the association between FluVac and dementia. Although our analyses adjusted for a wide range of potential confounders, including baseline and repeatedly measured factors, as well as environmental and genetic characteristics, we could not fully rule out residual confounding. The NCO analysis found no association between FluVac and hip fracture, indicating that the observed association was less likely caused by unmeasured confounders. Moreover, most of the lifestyle factors were self-reported, and some cases of dementia and PD were not recorded in the medical records or death registers. However, misclassification errors were likely to have biased the findings to the null and previous studies have established good agreement between dementia and PD case ascertainment and primary care records^25–27^. Besides, our analyses were restricted to UKB participants with primary care data, and the response rate of the UK Biobank survey was only 5.5%. Thus, the current cohort of participants might not be a representative sample of the UK population. Therefore, caution should be exercised when extrapolating the findings of the present study to other populations.

In conclusion, FluVac was significantly associated with a reduced risk of incident dementia but not PD in the UKB population. The association between FluVac and dementia was dose-dependent and thus stronger in people receiving multiple doses of vaccination. Further studies with more details of vaccine compositions in a higher representative population would provide more insights into the mechanism that underlies the effect of FluVac on dementia risk.

## Methods

### Study population

This study used data from the UKB, which recruited more than half a million participants of middle and old age across the United Kingdom in 2006–2010. All participants provided informed consent, completed touch-screen questionnaires and verbal interview, provided biological samples, and underwent physical examination^28^. The UKB got initial ethical approval from its own Ethics Advisory Committee (https://www.ukbiobank.ac.uk/ethics/).

We restricted our analyses to a subset of the entire cohort that could be linked to primary care data. This dataset contains variables that are considered the most important for epidemiological research, including coded clinical events, prescriptions, and a range of administrative codes^12^. Data on vaccination were available from England, Scotland, and Wales and were included in the primary care data. We further excluded individuals aged <60 years at baseline from the analysis as young people rarely develop dementia and PD. Comparisons between participants with and without primary care data are given in Supplementary Table 1. There were no significant differences between these two groups of participants in almost all characteristics except the distribution of assessment center.

### Exposure of influenza vaccine

Participant’s vaccination status was obtained from the GP events and GP prescription records in the primary care data by using Read v2, Read v3 (Clinical Terms Version 3), dm+d, and British National Formulary (BNF) codes. All of these codes for FluVac are given in the Supplementary Table 2 and Supplementary Table 3. The composition of influenza vaccines could be annually updated to best match the constantly changing influenza viruses, thus the elderly is recommended to get an annual influenza vaccine. Therefore, influenza vaccination status was defined as a time-varying exposure, which was measured in one-year intervals and before the start of each interval.

### Outcome definition and follow-up

The primary outcomes were incident all-cause dementia and PD, which were ascertained through an algorithm combining self-reported medical conditions, linked data from hospital admissions, death registries, and primary care data^29^. Incident cases were identified using Read v2, Read v3, and International Classification of Diseases 9th/10th (ICD-9/10) version codes, which are all given in the Supplementary Table 4. This algorithm has been validated and has a positive predictive value of 82.5% for all-cause dementia and 91% for PD^25–27^. We also defined three secondary outcomes including Alzheimer’s disease (AD), vascular dementia (VD), and all other dementias (OD) except AD and VD (Supplementary Table 4).

We defined the index date as the date participants first attending the assessment center for the baseline survey or one year after the first GP record, whichever occurred later. This would guarantee that the participants had at least one year of GP records before the index date. Follow-up started on one year after the index date until the first occurrence of one of the following events: diagnosis of dementia or PD, death, loss to follow-up, or the last date of hospital admission (30 September 2021 for England and Wales, and 24 September 2021 for Scotland). Thus, participants who had less than one year of follow-up were excluded, including those diagnosed with dementia or PD within one year of the index date. We also excluded participants who received influenza vaccine or had a diagnosis of dementia or PD before the index date (Supplementary Fig. 1) for identifying new receivers of FluVac and incident cases of dementia and PD.

### Covariates

Potential confounders were categorized as baseline and repeatedly measured covariates. All factors were collected at baseline through self-reported questionnaires, including sociodemographic characteristics (sex, age, education qualification, Townsend deprivation index (TDI), average household income, and region of assessment center), general health factors (self-reported health rating, family history of dementia/PD and body mass index [BMI]), mental health (mental health score), lifestyle (smoking and drinking status, diet, tea and coffee intake, physical activity, and social isolation). Further, the apolipoprotein E (ApoE) genotype was defined by the SNP rs429358 and rs7412. As ApoE ε4 is a well-recognized genetic risk factor for dementia^30^, we divided the study population into ApoE ε4 carriers (+/+ or +/−) and noncarriers (−/−).

Repeatedly measured factors were measured in a one-year interval lag before every influenza season, including FluVac invitation, comorbidities and medication use (aspirin, glucose-lowering agents, statins, calcium channel blockers (CCB), angiotensin-converting enzyme inhibitors [ACEI], angiotensin receptor blockers [ARB], proton-pump inhibitors [PPI], beta blocking agents (BBA), and diuretics).

Family history of dementia and PD was ascertained according to the illnesses of father, mother, and siblings. BMI was calculated using weight divided by height squared and was categorized as obesity (≥30.0 kg/m^2^), overweight (25.0 ~ 29.9 kg/m^2^), healthy weight (18.5 ~ 24.9 kg/m^2^), and underweight (<18.5 kg/m^2^). Mental health score was measured using 13 data fields related to mood and feeling according to a previous study^31^. Social isolation was defined based on the number of persons in the household, frequency of friend/family visits, and leisure/social activities and was further divided into least isolated, moderately isolated, or most isolated^32^. The consumption of at least four of seven commonly eaten food groups following recommendations on dietary priorities for cardiometabolic health was used to define a healthy diet^33^. Participants were considered to keep regular physical activity if they meet the recommendations of at least 150 min of moderate activity or 75 min of vigorous activity per week^33^. Comorbidities were measured using the Charlson comorbidity index (CCI), which included 16 classes of diseases. Commonly used medications and FluVac invitation were ascertained according to GP prescriptions and GP clinical events respectively. More details about the covariates are given in the Supplementary Tables 5 and 6.

### Statistical analyses

We first reported summary statistics of participants according to whether they received influenza vaccine or not in the study period. Missing values were treated as a separate category and frequency and percentage for categorical variables and mean and standard deviation for continuous covariates were calculated. Standardized mean difference (SMD) was calculated for comparisons of categorical and continuous factors. We applied a multivariable time-varying Cox regression to estimate the hazard ratios (HR) and 95% confidence intervals (CIs) of the association between incident dementia/PD and FluVac which was modeled as a time-varying variable. The full model was adjusted for all potential baseline and repeatedly measured confounders listed above including sociodemographic characteristics, general health factors, mental health, lifestyle, comorbidities and medication use. Robust sandwich-type variance estimators were applied to calculate confidence intervals and *p*-values as repeated measures of participants were used in the model.

### Secondary analysis

We assessed the associations between FluVac and the three secondary outcomes, including AD, VD, and OD. We next examined the association between FluVac and dementia/PD within different subgroups defined according to baseline characteristics for checking potential interactions: sex (female and male), age (60 ~ 64 and ≥65 years), education qualification (A levels or above and others), self-reported health rating (fair/poor and excellent/good), CCI (0 and ≥1), and ApoE4 gene type (ε4 carriers and noncarriers).

A dose-response relationship of the association was examined between the incidence of dementia/PD and cumulative FluVac, which was measured as the average number of influenza vaccinations per year (AvgFluVac) since the first vaccine dose. The AvgFluVac took a value in the interval of [0, 1] and a value approximating one indicated continuous vaccination while the value zero represented non-vaccinated status. We did not apply the total number of FluVac as the cumulative exposure because this measure could not differentiate between different patterns of vaccination that resulted in the same cumulative number^34^. For example, a participant who received five non-consecutive doses of FluVac would have the same total number as a participant who got vaccinated continuously for five years, regardless of how recently vaccination occurred. However, the AvgFluVac would change at different rates based on the vaccination pattern if participants were not vaccinated continuously. Then the AvgFluVac was modeled as a time-varying variate in two ways: categorical (0, ≤0.4, 0.41 ~ 0.6, 0.61 ~ 0.8, and >0.8) and continuous (restricted cubic spline functions with five knots at 0, 0.4, 0.6, 0.8, and 1 according to Desquilbet et al.^35^).

Because multiple comparisons in the analyses of subgroups, causal-specific dementia, and secondary analyses may increase the risk of type I error, findings of our secondary analyses should be interpreted as exploratory.

### Sensitivity analysis

Several sensitivity analyses were performed to examine the robustness of the results in the primary analysis. First, a marginal structural model (MSM) with time-varying inverse probability of treatment weighting (IPTW) and censoring weighting (IPCW) was applied to check the potential effects of time-varying confounding that was affected by previous exposure. Stabilized IPTW and IPCW were applied and potential selection bias by all-cause death was controlled using IPCW in the MSM. Second, the Fine-Gray subdistribution hazard model was fitted to check possible competing risk from all-cause mortality. Third, we used a washout period of two years and three years to define new receivers of FluVac, in which participants were required to have at least two or three years of GP records and be not vaccinated before the index date. Fourth, in the primary analyses, outcome cases that occurred within one year of the index date were excluded, in sensitivity analyses we further excluded dementia/PD cases diagnosed within two, three, and four years after the start of follow-up respectively, to eliminate possible prevalent cases and consequent causal inversion. Fifth, a small number of participants received live-attenuated influenza vaccine, which was mainly indicated for people aged 2 to 16 years and had different mechanisms of action from those of the intramuscular influenza vaccines^7^. For this, in sensitivity analyses we censored these participants at the time that they received a live-attenuated vaccine or excluded all of them from the final cohort. Sixth, we conducted a negative control outcome (NCO) analysis with hip fracture as the NCO to detect potential unmeasured confounding as no previous study has reported the association between FluVac and hip fracture. Thus, an association between FluVac and NCO would indicate the existence of unmeasured confounders.

All statistical analyses were performed using SAS 9.4 (SAS Institute Inc., Cary, NC, USA) and R version 4.2.1. *P* values were two-sided with statistical significance set at less than 0.05 (Figs. 1–3).

### Reporting summary

Further information on research design is available in the Nature Research Reporting Summary linked to this article.

### Supplementary information

Supplementary Information

REPORTING SUMMARY

## Data Availability

UK Biobank data are available online (https://www.ukbiobank.ac.uk/). All relevant data are available from the authors.

## Code availability

The code for this study is available in a ResearchGate repository (https://www.researchgate.net/publication/378011215_Code_FluVac_Dementia_PD).

## References

[1] Alzheimer’s Disease International, Guerchet M., Prince M., Prina M. *Numbers of people with dementia around the world: An update to the estimates in the World Alzheimer Report*, accessed 2 October 2023. https://www.alzint.org/resource/numbers-of-people-with-dementia-worldwide/ (2015).

[2] GBD 2016 Parkinson’s Disease Collaborators. Global, regional, and national burden of Parkinson’s disease, 1990–2016: a systematic analysis for the Global Burden of Disease Study 2016. *Lancet Neurol*. **17**, 939–953 (2018). 10.1016/S1474-4422(18)30295-3 PMC6191528 30287051

[3] GBD 2016 Dementia Collaborators. (2019). Global, regional, and national burden of Alzheimer’s disease and other dementias, 1990–2016: a systematic analysis for the Global Burden of Disease Study 2016. Lancet Neurol..

[4] Alzheimer’s Disease International. World Alzheimer Report 2018. London, accessed 2 October 2023. https://www.alzint.org/resource/world-alzheimer-report-2018/ (2018).

[5] Sipilä PN (2021). Hospital-treated infectious diseases and the risk of dementia: a large, multicohort, observational study with a replication cohort. Lancet Infect. Dis..

[6] Smeyne RJ, Noyce AJ, Byrne M, Savica R, Marras C (2021). Infection and risk of Parkinson’s disease. J. Parkinsons Dis..

[7] Pebody RG (2015). Uptake and impact of vaccinating school age children against influenza during a season with circulation of drifted influenza A and B strains, England, 2014/15. Eur. Surveill..

[8] Sun H, Liu M, Liu J (2023). Association of INfluenza Vaccination and Dementia Risk: A Meta-analysis of Cohort Studies. J. Alzheimers Dis..

[9] Veronese N (2022). Influenza vaccination reduces dementia risk: a systematic review and meta-analysis. Ageing Res. Rev..

[10] Torre, C. & Martins, A. P. Overview of Pharmacoepidemiological Databases in the Assessment of Medicines Under Real-Life Conditions. in *Epidemiology - Current Perspectives on Research and Practice* (ed Lunet, N.) (Rijeka: InTech, 2012).

[11] Lund JL, Richardson DB, Stürmer T (2015). The active comparator, new user study design in pharmacoepidemiology: historical foundations and contemporary application. Curr. Epidemiol. Rep..

[12] UK Biobank. UK Biobank Primary Care Linked Data Version 1.0, accessed 2 October 2023. https://biobank.ndph.ox.ac.uk/showcase/refer.cgi?id=591 (2019).

[13] Bukhbinder AS (2022). Risk of Alzheimer’s disease following influenza vaccination: a claims-based cohort study using propensity score matching. J. Alzheimers Dis..

[14] Wiemken TL (2021). Dementia risk following influenza vaccination in a large veteran cohort. Vaccine.

[15] Luo CS, Chi CC, Fang YA, Liu JC, Lee KY (2020). Influenza vaccination reduces dementia in patients with chronic obstructive pulmonary disease: a nationwide cohort study. J. Investig. Med..

[16] Lee CY (2020). Risk of dementia in patients with periodontitis and related protective factors: a nationwide retrospective cohort study. J. Clin. Periodontol..

[17] Liu JC (2016). Influenza vaccination reduces dementia risk in chronic kidney disease patients: a population-based cohort study. Medicine.

[18] Suissa S, Azoulay L (2012). Metformin and the risk of cancer: time-related biases in observational studies. Diabetes Care.

[19] Suissa S, Dell’Aniello S (2020). Time-related biases in pharmacoepidemiology. Pharmacoepidemiol. Drug Saf..

[20] Verreault R, Laurin D, Lindsay J, De Serres G (2001). Past exposure to vaccines and subsequent risk of Alzheimer’s disease. CMAJ.

[21] Wang H (2020). Bacterial, viral, and fungal infection-related risk of Parkinson’s disease: meta-analysis of cohort and case-control studies. Brain Behav..

[22] Cocoros NM (2021). Long-term risk of Parkinson disease following influenza and other infections. JAMA Neurol..

[23] Bukhbinder AS, Ling Y, Harris K, Jiang X, Schulz PE (2023). Do vaccinations influence the development of Alzheimer disease?. Hum. Vaccin Immunother..

[24] Xie A, Gao J, Xu L, Meng D (2014). Shared mechanisms of neurodegeneration in Alzheimer’s disease and Parkinson’s disease. Biomed. Res. Int..

[25] Wilkinson T (2019). Identifying dementia outcomes in UK Biobank: a validation study of primary care, hospital admissions and mortality data. Eur. J. Epidemiol..

[26] Bush K., Wilkinson T., Schnier C., Nolan J. & Sudlow C., on behalf of UK biobank outcome adjudication group. Definitions of dementia and the major diagnostic pathologies, UK Biobank phase 1 outcomes adjudication, accessed 2 October 2023. https://biobank.ndph.ox.ac.uk/ukb/ukb/docs/alg_outcome_pdp.pdf (2018).

[27] Bush K., Rannikmae K., Wilkinson T., Schnier C., Sudlow C., On behalf of UK Biobank Outcome Adjudication Group. Definitions of Parkinson’s Disease and the Major Causes of Parkinsonism, UK Biobank Phase 1 Outcomes Adjudication, accessed 2 October 2023 https://biobank.ndph.ox.ac.uk/ukb/ukb/docs/alg_outcome_pdp.pdf (2018).

[28] Sudlow C (2015). UK biobank: an open access resource for identifying the causes of a wide range of complex diseases of middle and old age. PLoS Med..

[29] UK Biobank Outcome Adjudication Group. Algorithmically defined outcomes (ADOs), accessed 2 October 2023. https://biobank.ndph.ox.ac.uk/showcase/refer.cgi?id=460 (2022).

[30] The Alzheimer’s Association. (2022). 2022 Alzheimer’s disease facts and figures. Alzheimers Dement..

[31] Hepsomali P, Groeger JA (2021). Diet, sleep, and mental health: insights from the UK Biobank study. Nutrients.

[32] Smith RW (2021). Social isolation and risk of heart disease and stroke: analysis of two large UK prospective studies. Lancet Public Health.

[33] Lourida I (2019). Association of lifestyle and genetic risk with incidence of dementia. JAMA.

[34] Danaei G, Rodríguez LAG, Cantero OF, Logan R, Hernán MA (2013). Observational data for comparative effectiveness research: an emulation of randomised trials of statins and primary prevention of coronary heart disease. Stat. Methods Med. Res..

[35] Desquilbet L, Mariotti F (2010). Dose-response analyses using restricted cubic spline functions in public health research. Stat. Med..

