# Supplementary material for: Prospective cohort study evaluating the association between influenza vaccination and neurodegenerative diseases
Source: NPJ Vaccines. 2024 Mar 2;9:51. doi: 10.1038/s41541-024-00841-z (PMC10908860; doi:10.1038/s41541-024-00841-z)
Supplement: Supplementary file 1 — Supplementary Information [file 41541_2024_841_MOESM1_ESM.pdf]

Table of Contents

Supplementary Figure 1. Flowchart of including participants from the UK Biobank..........................2
Supplementary Figure 2. Influenza vaccination in the study cohort..3
Supplementary Figure 3. Dose-response relationship of the association between influenza vaccination and risk of dementia or Parkinson's disease in the UK Biobank 4
Supplementary Table 1. Comparisons between participants with and without primary care data. 5
Supplementary Table 2. Read v2 codes and Read v3 codes used for identifying influenza vaccination in GP clinical data from the UK Biobank..8
Supplementary Table 3. Codes used for identifying influenza vaccination in the GP prescriptions data from the UK Biobank...11
Supplementary Table 4. Codes used for identifying dementia and Parkinson's disease 21
Supplementary Table 5. Definition of covariates..25
Supplementary Table 6. Comorbidities used to define Charlson comorbidity index 26
References...28

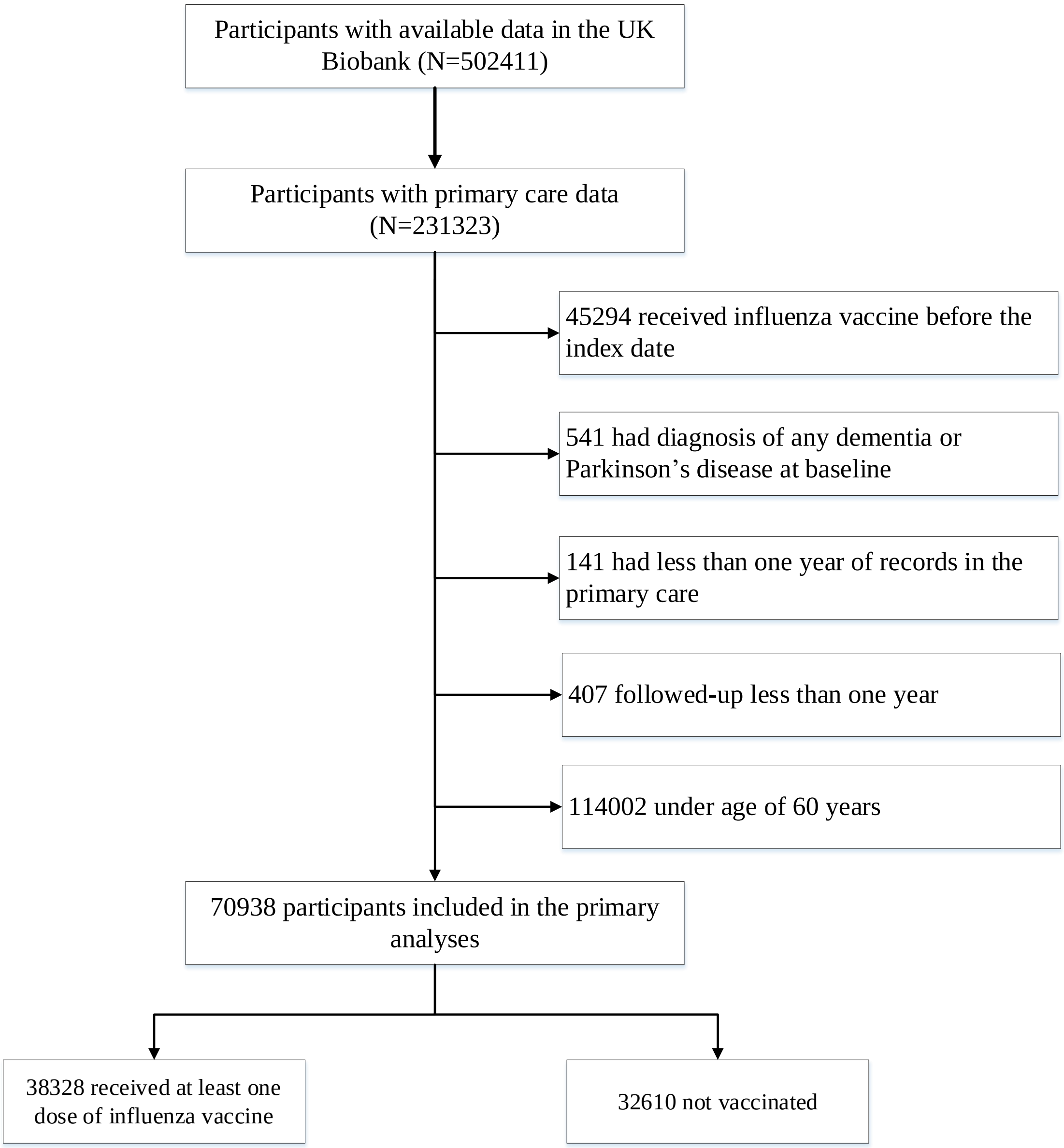

Supplementary Figure 1. Flowchart of including participants from the UK Biobank

(A)

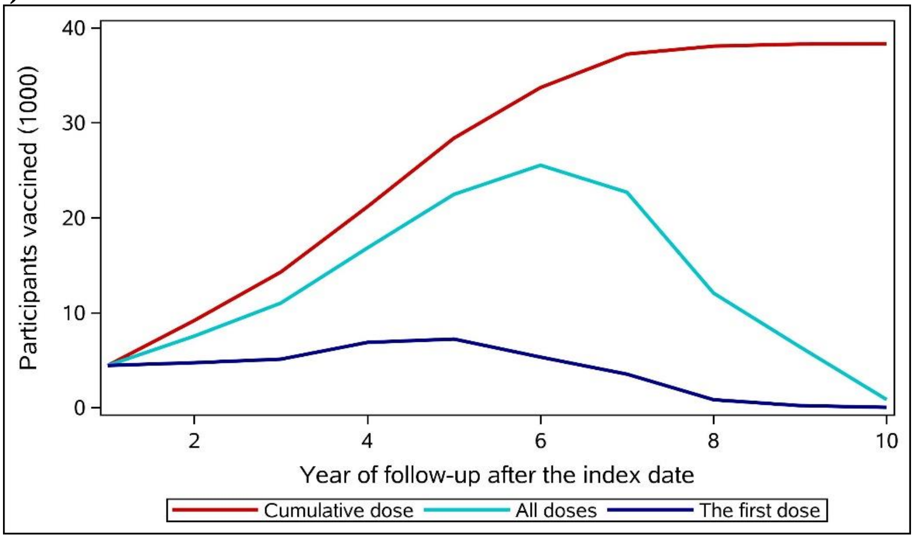

(B)

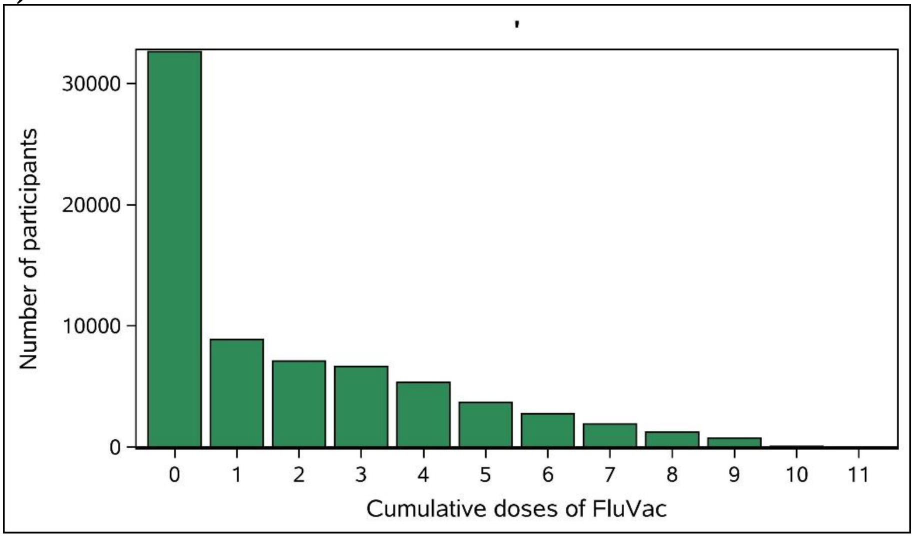

Supplementary Figure 2. Influenza vaccination in the study cohort.

(a) Trend of the number of participants receiving influenza vaccines after the index date. (b) Number of participants receiving different cumulative doses of influenza vaccinations.

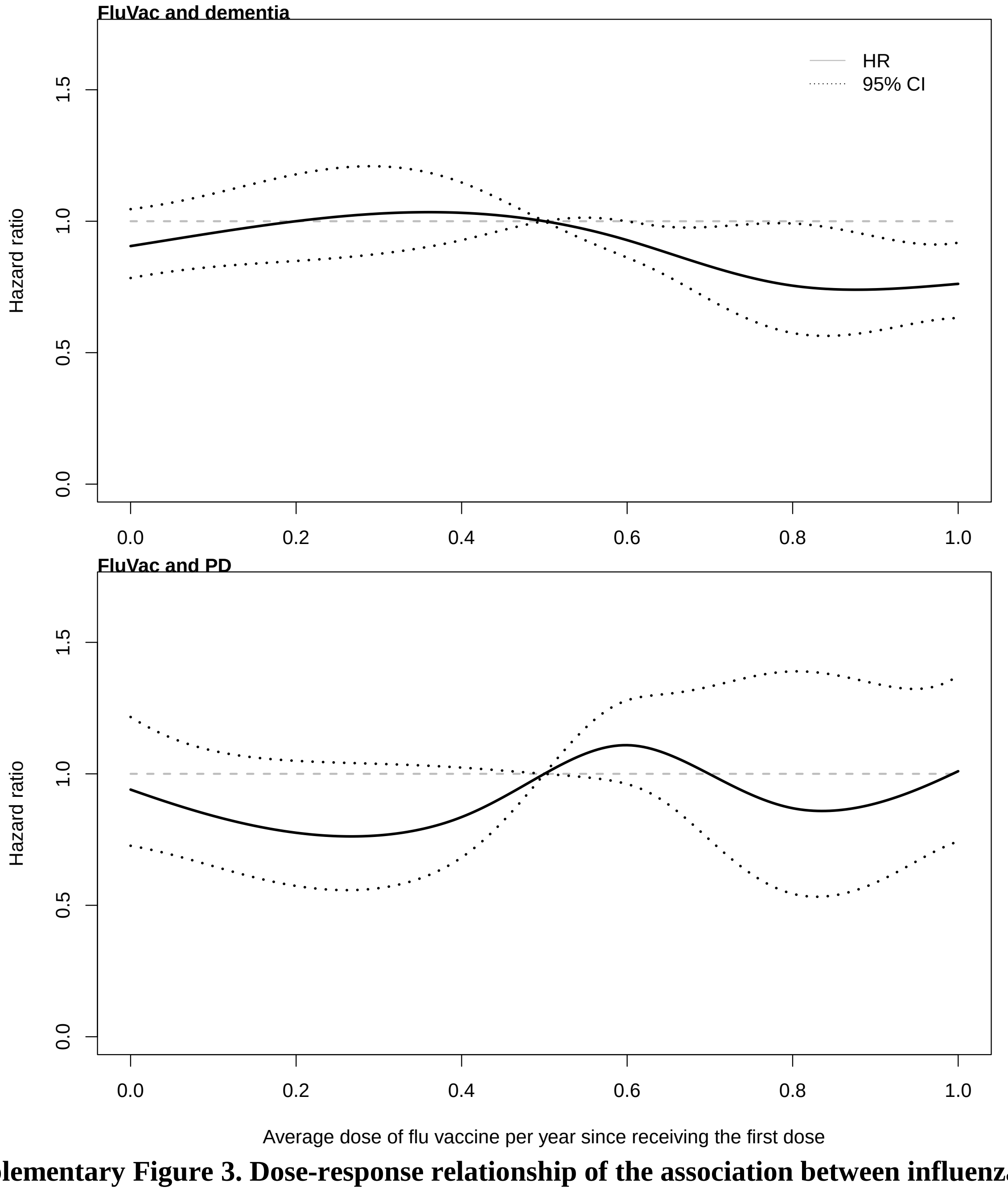

Supplementary Figure 3. Dose-response relationship of the association between influenza vaccination and risk of dementia or Parkinson's disease in the UK Biobank

*FluVac: influenza vaccination; PD: Parkinson's disease

Supplementary Table 1. Comparisons between participants with and without primary care data.

	Participants without primary care data (n=271079)	Participants with primary care data (n=231323)	SMD
**Male**, n(%)	124276 (45.8)	104806 (45.3)	0.011
**Age, mean (std)**	57.0 (8.11)	57.0 (8.06)	0.002
**Age group**, n(%)			
<50	63321 (23.4)	53869 (23.3)	0.002
50-54	40978 (15.1)	35163 (15.2)	0.002
55-59	48580 (17.9)	42003 (18.2)	0.006
60-64	65282 (24.1)	56072 (24.2)	0.004
⩾65	52915 (19.5)	44216 (19.1)	0.010
Unknown	3 (0.0)	0 (0.0)	0.005
**ApoE 4 gene type**			
ε4 negaitive	187181 (69.1)	161472 (69.8)	0.016
ε4 positive	74510 (27.5)	64000 (27.7)	0.004
Unknown	9388 (3.5)	5851 (2.5)	0.055
**Family history of dementia**, n(%)	31015 (11.4)	27405 (11.8)	0.013
**Family history of PD**, n(%)	10630 (3.9)	9295 (4.0)	0.005
**Education**, n(%)			
University/college degree	88854 (32.8)	73688 (31.9)	0.020
A levels/AS levels or equivalent	30824 (11.4)	24852 (10.7)	0.020
O-levels/GCEs/CSEs or equivalent	72262 (26.7)	60729 (26.3)	0.009
NVQ/HND/HNC or other professional qualification	30951 (11.4)	27826 (12.0)	0.019
Others	44637 (16.5)	41387 (17.9)	0.038
Unknown	3551 (1.3)	2841 (1.2)	0.007
**TDI**, mean (std)	-1.3 (3.15)	-1.3 (3.03)	0.023
**TDI**, n(%)			
≤-3.64	67739 (25.0)	57735 (25.0)	0.001
≤-2.14	67421 (24.9)	58019 (25.1)	0.005
≤0.55	66818 (24.6)	58603 (25.3)	0.016
>0.55	68819 (25.4)	56622 (24.5)	0.021
Unknown	282 (0.1)	344 (0.1)	0.013
**Average total household income**, n(%)			
Less than 18,000	51426 (19.0)	47889 (20.7)	0.043
18,000 to 30,999	57699 (21.3)	51297 (22.2)	0.022
31,000 to 51,999	60281 (22.2)	51342 (22.2)	0.001
Greater than 52,000	62305 (23.0)	47639 (20.6)	0.058
Unknown	39368 (14.5)	33156 (14.3)	0.005
**Study center region**, n (%)			
England	260049 (95.9)	185711 (80.3)	0.498
Wales	2397 (0.9)	18407 (8.0)	0.349
Scotland	8633 (3.2)	27205 (11.8)	0.331

**BMI**, mean (std)	27.2 (4.97)	27.4 (5.00)	0.037
**BMI**, n(%)			
<25	89484 (33.0)	72936 (31.5)	0.032
<18.5	1499 (0.6)	1135 (0.5)	0.009
<30	114181 (42.1)	97996 (42.4)	0.005
≥30	64341 (23.7)	57978 (25.1)	0.031
Unknown	1574 (0.6)	1278 (0.6)	0.004
**Smoking status**, n(%)			
Never	147084 (54.3)	126384 (54.6)	0.008
Previous	93605 (34.5)	79417 (34.3)	0.004
Current	28660 (10.6)	24302 (10.5)	0.002
Unknown	1730 (0.6)	1220 (0.5)	0.015
**Drinking status**, n(%)			
Never	11933 (4.4)	10447 (4.5)	0.006
Previous	9597 (3.5)	8497 (3.7)	0.007
Current	248551 (91.7)	211723 (91.5)	0.006
Unknown	998 (0.4)	656 (0.3)	0.015
**Health diet**, n(%)	76052 (28.1)	64629 (27.9)	0.003
**Tea intake**, n(%)			
0 cups	47432 (17.5)	41521 (17.9)	0.012
1-2 cup	62363 (23.0)	51887 (22.4)	0.014
3-4 cups	79016 (29.1)	67406 (29.1)	0.000
≥5 cups	80982 (29.9)	69601 (30.1)	0.005
Unknown	1286 (0.5)	908 (0.4)	0.012
**Coffee intake**, n(%)			
0 cups	79445 (29.3)	68036 (29.4)	0.002
1 cup	54642 (20.2)	45646 (19.7)	0.011
2 cups	51189 (18.9)	42634 (18.4)	0.012
≥3 cups	84473 (31.2)	74069 (32.0)	0.018
Unknown	1330 (0.5)	938 (0.4)	0.013
**Regular physic activity**, n(%)	213908 (78.9)	181813 (78.6)	0.008
**Self-health rating**			
Excellent	45158 (16.7)	36685 (15.9)	0.022
Good	156102 (57.6)	132861 (57.4)	0.003
Fair	55989 (20.7)	49350 (21.3)	0.017
Poor	11804 (4.4)	10965 (4.7)	0.019
Unknown	2026 (0.7)	1462 (0.6)	0.014
**Mental health score**, n(%)			
≤2	92139 (34.0)	76799 (33.2)	0.017
≤4	57964 (21.4)	48995 (21.2)	0.005
≤7	67798 (25.0)	58129 (25.1)	0.003
≥8	49385 (18.2)	44264 (19.1)	0.024
Unknown	3793 (1.4)	3136 (1.4)	0.004
**Social isolation**, n(%)			
Least isolated	118962 (43.9)	102983 (44.5)	0.013
Moderately isolated	107985 (39.8)	92054 (39.8)	0.001

Most isolated	35469 (13.1)	29404 (12.7)	0.011
Unknown	5016 (1.9)	3888 (1.7)	0.013
**Charlson comorbidity index**, n(%)			
0	186523 (68.8)	158766 (68.6)	0.004
1	49227 (18.2)	42388 (18.3)	0.004
2	23492 (8.7)	20119 (8.7)	0.001
>2	11837 (4.4)	10050 (4.3)	0.001

SMD: standardized mean difference; PD: Parkinson’s disease.

Supplementary Table 2. Read v2 codes and Read v3 codes used for identifying influenza vaccination in GP clinical data from the UK Biobank

Read v2 code	Read v3 code	Read code description
65E..		Influenza vaccination
65E0.		First pandemic influenza vaccination
65E1.		Second pandemic influenza vaccination
65E2.		Influenza vaccination given by other healthcare provider
65E20		Seasonal influenza vaccination given by other healthcare provider
65E21		First intranasal seasonal influenza vaccination given by other healthcare provider
65E22		Second intranasal seasonal influenza vaccination given by other healthcare provider
65E23		Second intramuscular seasonal influenza vaccination given by other healthcare provider
65E24		First intramuscular seasonal influenza vaccination given by other healthcare provider
65E3.		First pandemic influenza vaccination given by other healthcare provider
65E4.		Second pandemic influenza vaccination given by other healthcare provider
65E5.		CELVAPAN - first influenza A (H1N1v) 2009 vaccination given
65E6.		CELVAPAN - second influenza A (H1N1v) 2009 vaccination given
65E7.		CELVAPAN - first influenza A (H1N1v) 2009 vaccination given by other healthcare provider
65E8.		CELVAPAN - second influenza A (H1N1v) 2009 vaccination given by other healthcare provider
65E9.		PANDEMRIX - first influenza A (H1N1v) 2009 vaccination given
65EA.		PANDEMRIX - second influenza A (H1N1v) 2009 vaccination given
65EB.		PANDEMRIX - first influenza A (H1N1v) 2009 vaccination given by other healthcare provider
65EC.		PANDEMRIX - second influenza A (H1N1v) 2009 vaccination given by other healthcare provider
65ED.		Seasonal influenza vaccination
65ED0		Seasonal influenza vaccination given by pharmacist
65ED1		Administration of first intranasal seasonal influenza vaccination
65EE.		Administration of intranasal influenza vaccination
65EE0		Administration of first intranasal influenza vaccination
65EE1		Administration of second intranasal influenza vaccination
9OX..		Flu vaccination administration|Influenza vacc. administratn.
9OX1.		Has 'flu vaccination at home
9OX2.		Has'flu vaccination at surgery
9OX3.		Has 'flu vaccination at hosp.
9OX8.		Has influenza vaccination at work
9OXZ.		Influenza vacc.administrat.NOS
9k7..		Influenza immunisation for those in the 65 years and over and other at risk groups - enhanced services administration
ZV048		[V]Flu - influenza vaccination|[V]Influenza vaccination
	65E..	Influenza vaccination
	9OX..	Flu vaccination administration|Influenza vacc. administratn.
	9OX1.	Has 'flu vaccination at home|Has influenza vaccination at home
	9OX2.	Has influenza vaccination at surgery|Has'flu vaccination at surgery
	9OX3.	Has influenza vaccination at hospital
	9OXZ.	Influenza vacc.administrat.NOS

	XaK9t	Influenza immunisation for those in the 65 years and over and other at risk groups enhanced services administration
	XaKNY	Has influenza vaccination at work
	XaLK4	Booster influenza vaccination
	XaLNG	First pandemic influenza vaccination
	XaLNH	Second pandemic influenza vaccination
	XaPwi	First pandemic influenza vaccination given by other healthcare provider
	XaPwj	Second pandemic influenza vaccination given by other healthcare provider
	XaPyT	Influenza vaccination given by other healthcare provider
	XaQhk	CELVAPAN - first influenza A (H1N1v) 2009 vaccination given
	XaQhl	CELVAPAN - second influenza A (H1N1v) 2009 vaccination given
	XaQhm	PANDEMRIX - first influenza A (H1N1v) 2009 vaccination given
	XaQhn	PANDEMRIX - second influenza A (H1N1v) 2009 vaccination given
	XaQho	CELVAPAN - first influenza A (H1N1v) 2009 vaccination given by other healthcare provider
	XaQhq	PANDEMRIX - first influenza A (H1N1v) 2009 vaccination given by other healthcare provider
	XaQhr	PANDEMRIX - second influenza A (H1N1v) 2009 vaccination given by other healthcare provider
	XaZ0d	Seasonal influenza vaccination
	XaZ0e	Seasonal influenza vaccination given by other healthcare provider
	XaZfY	Seasonal influenza vaccination given by pharmacist
	Xaa9G	Administration of intranasal influenza vaccination
	XaaZp	Seasonal influenza vaccination given while hospital inpatient
	Xaac3	Administration of first intranasal seasonal influenza vaccination
	Xaac4	Administration of second intranasal seasonal influenza vaccination
	Xaac7	First intranasal seasonal influenza vaccination given by other healthcare provider
	Xaac8	Second intranasal seasonal influenza vaccination given by other healthcare provider
	XabvT	Second intramuscular seasonal influenza vaccination given by other healthcare provider
	Xac5J	First intramuscular seasonal influenza vaccination given by other healthcare provider
	Xad9j	Administration of first inactivated seasonal influenza vaccination
	ZV048	[V]Flu - influenza vaccination|[V]Influenza vaccination
	n47..	FLU - Influenza vaccine|Influenza vaccine
	n471.	Fluvirin prefilled syringe 0.5mL
	n473.	Influvac sub-unit prefilled syringe 0.5mL
	n477.	Inactivated Influenza vaccine injection 0.5mL
	n478.	Inactivated Influenza vaccine prefilled syringe 0.5mL
	n479.	Influenza vaccine Vials 5mL
	n47A.	PANDEMRIX INFLUENZA A VACCINE (H1N1v) 2009 injection
	n47D.	FLUENZ nasal suspension 0.2mL
	n47E.	INFLUENZA VACCINE (LIVE ATTENUATED) nasal suspension 0.2mL
	n47H.	FLUARIX TETRA suspension for injection prefill syringe 0.5mL
	n47I.	FLUENZ TETRA nasal spray suspension 0.2mL
	n47d.	Fluarix vaccine prefilled syringe
	n47e.	Begrivac vaccine pre-filled syringe 0.5mL
	n47f.	Agrippal vaccine prefilled syringe 0.5mL
	n47g.	Inactivated Influenza vaccine (split virion) prefilled syringe 0.5mL
	n47h.	Inactivated Influenza vaccine (surface antigen) prefilled syringe 0.5mL
	n47j.	MASTAFLU prefilled syringe 0.5mL

	n47m.	Enzira prefilled syringe 0.5mL
	n47n.	Viroflu prefilled syringe 0.5mL
	n47o.	IMUVAC prefilled syringe 0.5mL
	n47p.	INTANZA 15micrograms/strain susp for inj pfs 0.1mL
	n47q.	INACT INFLUENZA VACC (SPLIT VIRION) 15mcg/strain pfs 0.1mL
	n47t.	PANDEMRIX (H5N1) injection vials
	n47u.	INTANZA 9micrograms/strain susp for inj pfs 0.1mL
	n47v.	INACT INFLUENZA VACC (SPLIT VIRION) 9mcg/strain pfs 0.1mL
	n47y.	Inactivated Influenza vaccine (split virion) prefilled syringe 0.25mL
	n47z.	Inactivated Influenza vaccine (surface antigen virosome) prefilled syringe 0.5mL
	x006Z	Inactivated Influenza split virion vaccine
	x006a	Inactivated Influenza surface antigen sub-unit vaccine
	x00Yd	Fluvirin vaccine prefilled syringe
	x00Yi	Inactivated Influenza (split virion) vaccine prefilled syringe
	x00Yj	Inactivated Influenza (surface antigen sub-unit) vaccine prefilled syringe
	x00Yk	Influvac Sub-unit vaccine prefilled syringe
	x00Yp	MFV-Ject vaccine prefilled syringe
	x02d0	Fluarix
	x03qt	Begrivac vaccine prefilled syringe
	x03zt	Fluvirin
	x05Y1	Agrippal vaccine prefilled syringe
	x05oa	MASTAFLU prefilled syringe
	x05ob	MASTAFLU
	x05pi	Inflexal V
	x05yK	Enzira vaccine prefilled syringe
	x05yL	Enzira
	x05yP	Inactivated Influenza surface antigen virosome vaccine
	x05zC	Viroflu prefilled syringe
	x05zD	Viroflu

Supplementary Table 3. Codes used for identifying influenza vaccination in the GP prescriptions data from the UK Biobank

Read v2 code	dm+d code	BNF code	Drug name
n47..00	15651211000001104		Influenza H1N1 vaccine (split virion, inactivated, adjuva...
n47..00	15651211000001104		Influenza H1N1 vaccine (split virion, inactivated, adjuvante
n47..00	15651211000001104		Influenza H1N1 vaccine (split virion, inactivated, adjuvanted) emulsion and suspension for emulsion for injection
n471.			
n471.00	3249511000001109		FLUVIRIN 0.5 ML PRE-FILLED SYRINGE (EVANS)
n471.00	3249511000001109		FLUVIRIN 0.5 ml pre-filled syringe
n471.00	3249511000001109		FLUVIRIN 0.5 ml pre-filled syringe (Evans)
n471.00	3249511000001109		FLUVIRIN 0.5ml inj.
n471.00	3249511000001109		FLUVIRIN INJ
n471.00	3249511000001109		FLUVIRIN INJECTION
n471.00	3249511000001109		FLUVIRIN PRE-FILLED SYRINGE VAC 0.5
n471.00	3249511000001109		FLUVIRIN PRE-FILLED SYRINGE VAC 0.5ml
n471.00	3249511000001109		FLUVIRIN PRE-FILLED SYRINGE VAC 10.5ml
n471.00	3249511000001109		FLUVIRIN PRE-FILLED VAC 0.5
n471.00	3249511000001109		FLUVIRIN inj
n471.00	3249511000001109		FLUVIRIN injection
n471.00	3249511000001109		FLUVIRIN vaccine
n471.00	3249511000001109		Fluvirin Injection
n471.00	3249511000001109		Fluvirin Injection 0.5 ml pre-filled syringe
n471.00	3249511000001109		Fluvirin Injection
n471.00	3249511000001109		Fluvirin Injection 0.5 ml pre-filled syringe
n471.00	3249511000001109		Fluvirin injection
n471.00	3249511000001109		Fluvirin injection -
n471.00	3249511000001109		Fluvirin vaccine -
n471.00	3249511000001109		Fluvirin vaccine suspension for injection 0.5ml pre-fille...
n471.00	3249511000001109		Fluvirin vaccine suspension for injection 0.5ml pre-filled syringes (Novartis Vaccines and Diagnostics Ltd)
n473.	22628411000001107		Influvac Desu vaccine suspension for injection 0.5ml pre-...
n473.			
n473.00	22628411000001107		Influvac Desu vaccine suspension for injection 0.5ml pre-...
n473.00	3255011000001100		*INFLUENZA, VAC

n473.00	3255011000001100		INACTIVATED INFLUENZA VACCINE prefilled syringe 0.5mL
n473.00	3255011000001100		INFLUVAC DISPOSABLE SYRINGE VAC 0.5
n473.00	3255011000001100		INFLUVAC DISPOSABLE SYRINGE VAC 0.5ml
n473.00	3255011000001100		INFLUVAC DISPOSABLE VAC 0.5
n473.00	3255011000001100		INFLUVAC SUB-UNIT 0.5ml syringe
n473.00	3255011000001100		INFLUVAC SUB-UNIT inj
n473.00	3255011000001100		INFLUVAC SUB-UNIT prefilled syringe 0.5mL
n473.00	3255011000001100		INFLUVAC SUB-UNIT vaccine
n473.00	3255011000001100		INFLUVAC VAC
n473.00	3255011000001100		Influvac Sub-Unit Injection 0.5 ml pre-filled syringe
n473.00	3255011000001100		Influvac Sub-Unit Injection
n473.00	3255011000001100		Influvac Sub-Unit Injection 0.5 ml pre-filled syringe
n473.00	3255011000001100		Influvac Sub-unit vaccine suspension for injection 0.5ml ...
n473.00	3255011000001100		Influvac Sub-unit vaccine suspension for injection 0.5ml pre-filled syringes (BGP Products Ltd)
n473.00	3255011000001100		Influvac sub-unit vaccine -
n476.			
n476.00	170275001000027103		INFLUENZA (MERIEUX) 0.5ML PFS VAC
n476.00	170275001000027103		M.f.v.ject PERSONALLY ADMIN. SYRINGE 0.5 ML
n476.00	170275001000027103		MFV Ject Syringe 0.5ml
n476.00	170275001000027103		MFV-JECT 0.5 ml pre-filled syringe
n476.00	170275001000027103		MFV-JECT PRE-FILLED SYRINGE VAC 0.5ml
n476.00	170275001000027103		MFV-JECT PRE-FILLED VAC 0.5
n476.00	170275001000027103		MFV-JECT inj
n476.00	170275001000027103		MFV-JECT injection
n476.00	170275001000027103		Mfv-Ject Injection
n476.00	170275001000027103		Mfv-Ject Prefilled Syringe
n476.00	170275001000027103		Mfv-ject Vaccination (Aventis Pasteur MSD)
n476.00	170275001000027103		Mfv-ject Vaccine 0.5 ml syringe
n477.			
n477.00	348046004		INFLUENZA INACTIVATED SPLIT VIRION vaccine
n477.00	348046004		INFLUENZA INACTIVATED SPLIT VIRION vaccine [0.5
n477.00	348046004		INFLUENZA INACTIVATED SURFACE ANTIGEN INJ
n477.00	348046004		INFLUENZA VACCINE INJECTION
n477.00	348046004		Inactivated Influenza (Split Virion) Vaccine 0.5 ml pre...
n477.00	348046004		Inactivated Influenza (Split Virion) Vaccine 0.5 ml pre-fill

n477.00	348046004		Influenza inactivated split virion injection -
n477.00	348046004		Influenza inactivated split virion vaccine -
n477.00	348046004		Influenza vaccine (split virion, inactivated) suspension ...
n477.00	348046004		Influenza vaccine (split virion, inactivated) suspension for injection 0.5ml pre-filled syringes
n477.00	348046004		Influenza vaccine injection -
n478.			
n478.00	157905001000027100		INFLUENZA PRE-FILLED VAC
n47A.	15651011000001109		PANDEMRIX vaccine
n47A.	15651011000001109		Pandemrix (H1N1) Vaccine Emulsion For Injection
n47A.			
n47F.00	15382311000001101		Optaflu vaccine suspension for injection 0.5ml pre-filled...
n47H.		14040200	Fluarix Tetra vaccine suspension for injection 0.5ml pre-...
n47H.00	22704311000001109		Fluarix Tetra vaccine suspension for injection 0.5ml pre-...
n47a.			
n47b.00	69025001000027102		FLUZONE (PREFILLED S VAC
n47c.			
n47c.00	69025001000027102		FLUZONE (PREFILLED S VAC
n47c.00	69025001000027102		FLUZONE (PREFILLED SYRINGE) VAC
n47c.00	69025001000027102		FLUZONE influenza vaccine 0.5 ml syringe
n47c.00	69025001000027102		Fluzone Vaccine 0.5 ml
n47d.		14040200	Fluarix vaccine suspension for injection 0.5ml pre-filled...
n47d.			
n47d.00	3245911000001104		FLUARIX INJECTION
n47d.00	3245911000001104		FLUARIX PRE-FILLED VAC
n47d.00	3245911000001104		FLUARIX inj
n47d.00	3245911000001104		FLUARIX injection
n47d.00	3245911000001104		FLUARIX vaccine
n47d.00	3245911000001104		Fluarix Vaccine
n47d.00	3245911000001104		Fluarix vaccine suspension for injection 0.5ml pre-filled...
n47e.			
n47e.00	3247011000001105		BEGRIVAC VAC
n47e.00	3247011000001105		BEGRIVAC VACCINE
n47e.00	3247011000001105		BEGRIVAC vaccine
n47e.00	3247011000001105		BEGRIVAC vaccine [0.5ml pre-fill syrng]
n47e.00	3247011000001105		Begrivac Vaccine

n47e.00	3247011000001105		Begrivac vaccine -
n47f.			
n47f.00	3255311000001102		AGRIPPAL vaccine
n47f.00	3255311000001102		Agrippal Injection
n47f.00	3255311000001102		Agrippal Injection
n47f.00	3255311000001102		Agrippal vaccine -
n47f.00	3255311000001102		Agrippal vaccine suspension for injection 0.5ml pre-fille...
n47f.00	3255311000001102		Agrippal vaccine suspension for injection 0.5ml pre-filled s
n47f.00	3255311000001102		Agrippal vaccine suspension for injection 0.5ml pre-filled syringes (Seqirus Ltd)
n47g.		14040200	Influenza vaccine (split virion inactivated) suspension ...
n47g.			
n47g.00	11278411000001109		Influenza vaccine (split virion, inactivated) suspension for
n47g.00	3244411000001106		Influenza vaccine (split virion, inactivated) suspension ...
n47g.00	3244411000001106		Influenza vaccine (split virion, inactivated) suspension for
n47g.00	3244411000001106		Sanofi Pasteur Msd Inactivated Influenza Split Virion Vacci
n47g.00	348046004		INFLUENZA INACTIVATED SPLIT VIRION vaccine
n47g.00	348046004		Inactivated Influenza (Split Virion) Vaccine 0.5 ml pre-fi
n47g.00	348046004		Inactivated Influenza (Split Virion) Vaccine 0.5 ml pre...
n47g.00	348046004		Influenza inactivated split virion vaccine -
n47g.00	348046004		Influenza vaccine (split virion, inactivated) suspension ...
n47g.00	348046004		Influenza vaccine (split virion, inactivated) suspension for
n47g.00	348046004		Influenza vaccine (split virion, inactivated) suspension for injection 0.5ml pre-filled syringes
n47g.00	348046004		influenza inactivated split virion vaccine
n47h.		14040200	Influenza vaccine (surface antigen inactivated) suspensi...
n47h.			
n47h.00	157905001000027100		INFLUENZA INACTIVATED SURFACE ANTIGEN inj
n47h.00	157905001000027100		INFLUENZA INACTIVATED SURFACE ANTIGEN injection
n47h.00	157905001000027100		INFLUENZA INACTIVATED SURFACE ANTIGEN vaccine
n47h.00	157905001000027100		INFLUENZA PRE-FILLED SYRINGE VAC
n47h.00	157905001000027100		INFLUENZA PRE-FILLED VAC
n47h.00	157905001000027100		INFLUENZA VAC
n47h.00	157905001000027100		INFLUENZA VACCIN INACTIVATE SURFAC ANTIGE INJ
n47h.00	157905001000027100		Inactivated Influenza Vaccine, Surface Antigen Injection 0
n47h.00	157905001000027100		Inactivated Influenza Vaccine, Surface Antigen Injection 0.5
n47h.00	157905001000027100		Inactivated influenza Vaccine 0.5 ml in preloaded syringe

n47h.00	157905001000027100		Influenza Vaccine N/a
n47h.00	157905001000027100		Influenza inactivated surface antigen Vaccination
n47h.00	157905001000027100		Influenza inactivated surface antigen injection -
n47h.00	157905001000027100		Influenza inactivated surface antigen vaccine -
n47h.00	157905001000027100		Influenza vaccine inactivated surface antigen injection -
n47h.00	348047008		Inactivated Influenza Vaccine, Surface Antigen Injection 0
n47h.00	348047008		Inactivated Influenza Vaccine, Surface Antigen Injection...
n47h.00	348047008		Inactivated Influenza Vaccine, Surface Antigen Injection 0.5
n47h.00	348047008		Influenza (Split Virion, Inactivated) Vaccine 15 microgram s
n47h.00	348047008		Influenza vaccine (surface antigen, inactivated) suspensi...
n47h.00	348047008		Influenza vaccine (surface antigen, inactivated) suspension
n47h.00	348047008		Influenza vaccine (surface antigen, inactivated) suspension for injection 0.5ml pre-filled syringes
n47i.			
n47i.00	10306611000001101		INFLEXAL V vaccine
n47j.			
n47j.00	4365711000001104		MASTAFLU vaccine
n47k.			
n47k.00	10306611000001101		INFLEXAL V vaccine
n47l.00	10306411000001104		INVIVAC vaccine
n47m.			
n47m.00	9511411000001107		ENZIRA vaccine
n47m.00	9511411000001107		Enzira Suspension For Injection 0.5 ml pre-filled syringe
n47m.00	9511411000001107		Enzira Suspension For Injection 0.5 ml pre-filled syringe
n47m.00	9511411000001107		Enzira vaccine -
n47m.00	9511411000001107		Enzira vaccine suspension for injection 0.5ml pre-filled ...
n47m.00	9511411000001107		Enzira vaccine suspension for injection 0.5ml pre-filled syr
n47m.00	9511411000001107		Enzira vaccine suspension for injection 0.5ml pre-filled syringes (Pfizer Ltd)
n47n.			
n47n.00	10455811000001107		VIROFLU vaccine
n47n.00	10455811000001107		Viroflu Suspension For Injection 0.5 ml pre-filled syringe
n47o.			
n47o.00	10859911000001105		IMUVAC vaccine
n47o.00	10859911000001105		Imuvac Suspension For Injection 0.5 ml pre-filled syringe
n47o.00	10859911000001105		Imuvac Suspension For Injection 0.5 ml pre-filled syringe
n47o.00	10859911000001105		Imuvac vaccine -

n47o.00	10859911000001105		Imuvac vaccine suspension for injection 0.5ml pre-filled ...
n47o.00	10859911000001105		Imuvac vaccine suspension for injection 0.5ml pre-filled syr
n47p.			
n47p.00	15506511000001107		INTANZA vaccine 15micrograms
n47p.00	15506511000001107		Intanza 15microgram strain vaccine suspension for injecti...
n47q.			
n47q.00	15507511000001109		INFLUENZA INACTIVATED SPLIT VIRION vaccine 15micrograms
n47q.00	15507511000001109		Influenza (Split Virion, Inactivated) Vaccine 15 microg...
n47q.00	15507511000001109		Influenza (Split Virion, Inactivated) Vaccine 15 microgram
n47q.00	15507511000001109		Influenza vaccine (split virion, inactivated) 15microgram...
n47u.00	15454511000001101		INTANZA vaccine 9micrograms
n47v.			
n47v.00	15474011000001107		INFLUENZA INACTIVATED SPLIT VIRION vaccine 9micrograms
n47z.			
n47z.00	10309511000001105		Inactivated Influenza Vaccine, Surface Antigen, Virosome Su
		14.04.09.00.00	Agrippal vaccine suspension for injection 0.5ml pre-filled syringes (Novartis Vaccines and Diagnostics Ltd)
		14.04.09.00.00	Begrivac vaccine suspension for injection 0.5ml pre-filled syringes (Novartis Vaccines and Diagnostics Ltd)
		14.04.09.00.00	Celvapan (H1N1) vaccine (whole virion, Vero cell derived, inactivated) suspension for injection (Baxter Healthcare Ltd)
		14.04.09.00.00	Enzira vaccine suspension for injection 0.5ml pre-filled syringes (Pfizer Ltd)
		14.04.09.00.00	FLUZONE vaccine [AV/PASTEUR]
		14.04.09.00.00	Fluarix Tetra vaccine suspension for injection 0.5ml pre-filled syringes (GlaxoSmithKline UK Ltd)
		14.04.09.00.00	Fluarix vaccine suspension for injection 0.5ml pre-filled syringes (GlaxoSmithKline UK Ltd)
		14.04.09.00.00	Fluenz vaccine nasal suspension 0.2ml unit dose (AstraZeneca UK Ltd)
		14.04.09.00.00	Fluvirin vaccine suspension for injection 0.5ml pre-filled syringes (Novartis Vaccines and Diagnostics Ltd)
		14.04.09.00.00	INFLUENZA INACTIVATED SPLIT VIRION vaccine [AV/PASTEUR]
		14.04.09.00.00	INFLUENZA INACTIVATED SPLIT VIRION vaccine [CHIRON]
		14.04.09.00.00	INFLUENZA INACTIVATED SPLIT VIRION vaccine [SAN PAST]
		14.04.09.00.00	Imuvac vaccine suspension for injection 0.5ml pre-filled syringes (BGP Products Ltd)
		14.04.09.00.00	Inflexal V vaccine suspension for injection 0.5ml pre-filled syringes (Janssen-Cilag Ltd)

		14.04.09.00.00	Influenza H1N1 vaccine (split virion, inactivated, adjuvanted) emulsion and suspension for emulsion for injection
		14.04.09.00.00	Influenza vaccine (live attenuated) nasal suspension 0.2ml unit dose
		14.04.09.00.00	Influenza vaccine (split virion, inactivated) 15microgram strain suspension for injection 0.1ml pre-filled syringes
		14.04.09.00.00	Influenza vaccine (split virion, inactivated) 9microgram strain suspension for injection 0.1ml pre-filled syringes
		14.04.09.00.00	Influenza vaccine (split virion, inactivated) suspension for injection 0.5ml pre-filled syringes
		14.04.09.00.00	Influenza vaccine (split virion, inactivated) suspension for injection 0.5ml pre-filled syringes (Pfizer Ltd)
		14.04.09.00.00	Influenza vaccine (split virion, inactivated) suspension for injection 0.5ml pre-filled syringes (sanofi pasteur MSD Ltd)
		14.04.09.00.00	Influenza vaccine (surface antigen, inactivated) suspension for injection 0.5ml pre-filled syringes (Novartis Vaccines and Diagnostics Ltd)
		14.04.09.00.00	Influvac Desu vaccine suspension for injection 0.5ml pre-filled syringes (Abbott Healthcare Products Ltd)
		14.04.09.00.00	Influvac Sub-unit vaccine suspension for injection 0.5ml pre-filled syringes (BGP Products Ltd)
		14.04.09.00.00	Intanza 15microgram strain vaccine suspension for injection 0.1ml pre-filled syringes (sanofi pasteur MSD Ltd)
		14.04.09.00.00	Intanza 9microgram strain vaccine suspension for injection 0.1ml pre-filled syringes (sanofi pasteur MSD Ltd)
		14.04.09.00.00	Invivac vaccine suspension for injection 0.5ml pre-filled syringes (Abbott Healthcare Products Ltd)
		14.04.09.00.00	MFV-JECT vaccine [AV/PASTEUR]
		14.04.09.00.00	Mastaflu vaccine suspension for injection 0.5ml pre-filled syringes (Masta Ltd)
		14.04.09.00.00	Optaflu vaccine suspension for injection 0.5ml pre-filled syringes (Novartis Vaccines and Diagnostics Ltd)
		14.04.09.00.00	Pandemrix vaccine emulsion and suspension for emulsion for injection (GlaxoSmithKline UK Ltd)
		14.04.09.00.00	Viroflu vaccine suspension for injection 0.5ml pre-filled syringes (Janssen-Cilag Ltd)
		14.04.09.00.00	influenza inactivated split virion paediatric vaccine
		14.04.09.00.00	influenza inactivated surface antigen (prepared in cell cultures) vaccine
		14.04.09.00.00	influenza inactivated surface antigen vaccine
		1404	Imuvac Suspension For Injection 0.5 ml pre-filled syringe

		1404	Influenza (Split Virion Inactivated) Vaccine 15 microgram strain 0.1 ml pre-filled syringe
		1404	Influenza (Split Virion Inactivated) Vaccine 9 microgram strain 0.1 ml pre-filled syringe
		1404000H0AAAFAF	Inactivated Influenza (Split Virion) Vaccine 0.5 ml pre-filled syringe
		1404000H0AAAFAF	Inactivated Influenza Vaccine Surface Antigen Injection 0.5 ml pre-filled syringe
		1404000H0BBAAAF	Fluvirin Injection
		1404000H0BBAAAF	Fluvirin Syringe Gp10a 0.5ml VACC
		1404000H0BCAAAF	INFLUVAC SUB UNIT SYRINGE GP10 0.5ML VACC
		1404000H0BCAAAF	INFLUVAC VACC
		1404000H0BCAAAF	Influvac Sub Unit Syringe Gp10 0.5ml VACC
		1404000H0BCAAAF	Influvac Sub Unit Syringe Gp10a 0.5ml VACC
		1404000H0BCAAAF	Influvac Sub-Unit Injection
		1404000H0BCAAAF	Influvac Sub-Unit Injection 0.5 ml pre-filled syringe
		1404000H0BDAAAF	Pasteur Merieux Inactivated Influenza Vaccine 0.5 ml
		1404000H0BFAAAF	Fluarix Syringe Gp10a 0.5ml VACC
	116105001000027105		FLU VACCINE
	116105001000027105		Influenza inactivated split virion injection -
	116105001000027105		Influenza inactivated split virion vaccine -
	116105001000027105		Sanofi Pasteur Msd Inactivated Influenza Split Virion Vaccin
	148265001000027104		FLUVIRIN AQUEOUS VAC
	15651211000001104		INFLUENZA (H1N1) INACTIVATED SPLIT VIRION vaccine
	222865001000027100		INACTIVTD INFLUENZA SPLIT VIRION MERIEUX VAC
	222865001000027100		INACTIVTD INFLUENZA VAC
	222865001000027100		INFLUENZA INACTIVATED SPLIT VIRION inj
	222865001000027100		INFLUENZA INACTIVATED SPLIT VIRION vaccine
	222865001000027100		Influenza inactivated split virion Vaccination (Aventis Pasteur MSD)
	299275001000027104		AGRIPPAL 0.5ML PRE-FILLED SYRI
	299275001000027104		BEGRIVAC P/FILLED SYRINGE 0.5ML
	299275001000027104		BEGRIVAC P/FILLED SYRINGE 0.5ML[0.5ml pre-fillsyrng]
	299275001000027104		BEGRIVAC vaccine [0.5ml pre-fill syrng]
	299275001000027104		Begrivac Vaccine
	299275001000027104		DUPHAR INFLUVAC 0.5ML
	299275001000027104		FLU VACCINE SPLIT VIRION
	299275001000027104		FLU/VAC/SPLIT VIRION (MERIEUX) PRE-FILLED SYRINGE
	299275001000027104		FLU/VAC/SPLIT prefilled syringe (Merieux)
	299275001000027104		FLUARIX INJ

	299275001000027104		FLUVIRIN
	299275001000027104		FLUVIRIN 0.5 ml pre-filled syringe (Evans)
	299275001000027104		FLUVIRIN INJ
	299275001000027104		FLUVIRIN INJECTION
	299275001000027104		FLUVIRIN PREFILLED SYRINGE
	299275001000027104		FLUVIRIN VAC O.5ML PF SYRG E5285AB1
	299275001000027104		FLUVIRIN injection
	299275001000027104		FLUVIRIN injection [0.5ml syringe(s)]
	299275001000027104		FLUVIRIN prefilled syringe 0.5mL
	299275001000027104		FLUZONE INJECTION
	299275001000027104		FLUZONE VAC 0.5ML PF SYRG
	299275001000027104		FLUZONE influenza vaccine 0.5 ml syringe
	299275001000027104		FLUZONE prefilled syringe 0.5mL
	299275001000027104		Fluarix Vaccine
	299275001000027104		Fluarix Vaccine 0.5 ml pre-filled syringe
	299275001000027104		Fluvirin Injection
	299275001000027104		Fluvirin Vaccine 0.5 ml syringe
	299275001000027104		Fluzone Pre-filled syringe N/A
	299275001000027104		Fluzone Vaccine 0.5 ml
	299275001000027104		INFLEXAL BERNA V vaccine [0.5ml pre-fill syrng]
	299275001000027104		INFLEXAL V 0.5ML PREFILLED SYRINGE
	299275001000027104		INFLU.VAC.(SPLIT VIRION)
	299275001000027104		INFLUENZA (MERIEUX) VAC INACT 0.5ML PFS
	299275001000027104		INFLUENZA INJECTION
	299275001000027104		INFLUENZA VAC INACTIVATED 0.5ML SYRG
	299275001000027104		INFLUENZA VACC 0.5ml syringe
	299275001000027104		INFLUENZA VACCINE INACTIVATED 0.5 ml pre-filled syringe
	299275001000027104		INFLUENZA VACCINE prefilled syringe 0.5mL
	299275001000027104		INFLUVAC
	299275001000027104		INFLUVAC SUB-UNIT 0.5 ml pre-filled syringe
	299275001000027104		INFLUVAC SUB-UNIT injection
	299275001000027104		Inactivated Influenza Vaccine, Surface Antigen Injection 0
	299275001000027104		Inactivated Influenza Vaccine, Surface Antigen Injection 0.5
	299275001000027104		Influenza Vac Evans.
	299275001000027104		Influenza Vacc Evans
	299275001000027104		Influenza Vaccination/FLUVARIN

	299275001000027104		Influenza Vaccine XXXXXXX
	299275001000027104		Influenza inactivated split virion [MERIEUX] injection -
	299275001000027104		Influenza inactivated surface antigen injection -
	299275001000027104		Influenza vacc FLUVARIN
	299275001000027104		Influenza vaccination
	299275001000027104		Influenza vaccination(FLUVIRIN
	299275001000027104		Influenza vaccine Pasteur merieux K5588 Syringe
	299275001000027104		Influvac Sub-Unit Injection
	299275001000027104		Influvac sub-unit injection -
	299275001000027104		Influvac sub-unit vaccine -
	299275001000027104		M.f.v. ject influenza vaccine SINGLE DOSE SYRINGE 0.5 ML
	299275001000027104		MERIEUX FLU VACC PRE-FILLED INJ 0.5ML
	299275001000027104		MERIEUX FLU VACCINE PREFILLED injection 0.5 ml.
	299275001000027104		MFV Ject Syringe 0,5ml
	299275001000027104		Mastaflu vaccine -
	299275001000027104		Mfv ject Syringe 0.5mls
	299275001000027104		Mfv-ject Vaccine 0.5 ml syringe
	299275001000027104		Mfv-ject injection -
	299275001000027104		SOLVAY INFLUVAC PFS
	3252611000001103		Influenza inactivated surface antigen vaccine -
	98025001000027103		FLU/VAC/SPLIT-MERIEUX PREFILLED SYRINGE VAC

Supplementary Table 4. Codes used for identifying dementia and Parkinson's disease

Outcomes	ICD-9 code	ICD-10 code	Self-reported disease code	Read 2 code	Read 3 code
All-cause dementia	331.0, 290.4, 331.1, 290.2, 290.3, 291.2, 294.1, 331.2, 331.5	F00, F00.0, F00.1, F00.2, F00.9, G30, G30.0, G30.1, G30.8, G30.9, F01, F01.0, F01.1, F01.2, F01.3, F01.8, F01.9, I67.3, F02.0, G31.0, A81.0, F02, F02.1, F02.2, F02.3, F02.4, F02.8, F03, F05.1, F10.6, G31.1, G31.8	1263	1461,38C13,3AE3.,3AE4.,3AE5.,3AE6.,66h..,6AB..,8BM02,8BM50,8BM60,8BPa.,8CMG2,8CMZ.,8CMZ0,8CMZ1,8CMZ2,8CMZ3,8CMe0,8CSA.,8Hla.,8IAe0,8IAe2,9Ou..,9Ou1.,9Ou2.,9Ou3.,9Ou4.,9Ou5.,9hD..,9hD0.,9hD1.,A411.,A4110,E00..,E000.,E001.,E0010,E0011,E0012,E0013,E001z,E002.,E0020,E0021,E002z,E003.,E004.,E0040,E0041,E0042,E0043,E004z,E012.,E02y1,E041.,Eu00.,Eu000,Eu001,Eu002,Eu00z,Eu01.,Eu010,Eu011,Eu012,Eu013,Eu01y,Eu01z,Eu02.,Eu020,Eu021,Eu022,Eu023,Eu024,Eu025,Eu02y,Eu02z,Eu041,Eu106,Eu107,F110.,F1100,F1101,F111.,F112.,F116.,F118.,F11x2,F11x7,F11x9,F11y2,F21y2,Fyu30	.1461,.3AE3,.3AE4,.3AE5,.3AE6,.66h.,.6AB.,.9Ou.,.9Ou1,.9Ou2,.9Ou3,.9Ou4,.9Ou5,.9hD1,.E11.,.E111,.E112,.E113,.E114,.E115,.E116,.E11Z,.F21Z,.F371,.G78.,1461,3AE3.,3AE4.,3AE5.,3AE6.,66h..,6AB..,8BM02,8BM50,8BPa.,8CMG2,8CMZ.,8CMZ0,8CMZ1,8CMZ2,8CMZ3,8CMe0,8CSA.,8IAe0,8IAe2,9Ou.,9Ou1.,9Ou2.,9Ou3.,9Ou4.,9Ou5.,9hD1.,A411.,A4110,E00..,E000.,E001.,E0010,E0011,E0012,E0013,E001z,E002.,E0020,E0021,E002z,E003.,E004.,E0040,E0041,E0042,E0043,E004z,E012.,E02y1,E041.,Eu00.,Eu000,Eu001,Eu002,Eu00z,Eu01.,Eu010,Eu011,Eu012,Eu013,Eu01y,Eu01z,Eu02.,Eu020,Eu021,Eu022,Eu023,Eu024,Eu025,Eu02y,Eu02z,Eu041,F110.,F1100,F1101,F111.,F112.,F116.,F118.,F11x2,F11x7,F11y2,F21y2,Fyu30,Ub1T6,X002m,X002w,X002x,X002y,X002z,X0030,X0031,X0032,X0033,X0034,X0035,X0036,X0037,X0039,X003A,X003B,X003C,X003D,X003E,X003F,X003G,X003H,X003I,X003J,X003P,X003R,X003T,X003V,X003W,X003X,X003l,X003m,X00R2,X00Rk,XE17j,XE1Xs,XE1Xu,XE1Z6,XE1aG,Xa0fZ,Xa0lH,Xa0sC,Xa0sE,Xa1GB,Xa25J,Xa3ez,Xa

					A1S,XaE74,XaIKB,XaIKC,XaJBQ,XaJBU,XaJBV,XaJBW,XaJBX,XaJPy,XaKyY,XaLFf,XaLFo,XaLFp,XaMFy,XaMG0,XaMGF,XaMGG,XaMGI,XaMGJ,XaMGK,XaMJC,XaOfZ,XaYFR,XaYPX,XaZWz,XaZqJ,XaaBZ,XaaeA,XaaiW,XabEk,XabEl,XabVp,Xabd2,Xabd3,XabtQ,XacIx,XacIy,XacIz,XacJ0,XacLx,XacM2,Xacly,Xaclz,Xaefu
Alzheimer's disease	331.0	F00, F00.0, F00.1, F00.2, F00.9, G30, G30.0, G30.1, G30.8, G30.9		Eu00.,Eu000,Eu001,Eu002,Eu00z,F110.,F1100,F1101,Fyu30	.F21Z,Eu00.,Eu000,Eu001,Eu002,Eu00z,F110.,F1100,F1101,Fyu30,X002x,X002y,X002z,X0030,X0031,X0032,X0033,X003G,XE17j,XaIKB,XaIKC
Vascular dementia	290.4	F01, F01.0, F01.1, F01.2, F01.3, F01.8, F01.9		E004.,E0040,E0041,E0042,E0043,E004z,E012.,Eu01.,Eu010,Eu011,Eu012,Eu013,Eu01y,Eu01z,F11x2,F21y2	.E115,.E116,.G78.,E004.,E0040,E0041,E0042,E0043,E004z,Eu01.,Eu010,Eu011,Eu012,Eu013,Eu01y,Eu01z,F11x2,F21y2,X003R,X003T,X003V,X003W,XE1Xs,Xa0lH
Parkinson's disease	3320,3321,3330	G20,G21,G21.0,G21.1,G21.2,G21.3,G21.4,G21.8,G21.9,G22,G23.0,G23.1,G23.2,G23.3,G23.8,G23.9,G25.9,G26,G90.3	1262	147F.,8Hx0.,8T06.,8T060,9Nle.,A94y1,Eu023,F11x9,F11y2,F12..,F120.,F121.,F122.,F123.,F124.,F12W.,F12X.,F12z.,F1302,F1303,F1304,F130z,F13z0,F13z3,F13zz,F174.,F1740,F1741,F24y0,F24y2,Fyu20,Fyu21,Fyu22,Fyu29,Fyu2B,d171.,dq...,dq1..,dq11.,dq12.,dq13.,dq14.,dq15.,dq16.,dq17.,dq18.,dq2.,dq21.,dq22.,dq23.,dq24.,dq25.,dq26.,dq27.,dq28.,dq29.,dq2a.,dq2b.,dq2c.,dq3..,dq31.,dq32.,dq33.,dq34.,dq35.,dq36.,dq37.,dq38.,dq39.,dq3A.,dq3B.,dq3C.,dq3D.,dq3E.,dq3F.,dq3G.,dq3H.,dq3I,dq3J.,	.F22.,.F221,.F222,.F22Z,147F.,8Hx0.,8T06.,8T060,A94y1,Eu023,F11x9,F11y2,F12..,F120.,F121.,F123.,F124.,F12W.,F12X.,F12z.,F1302,F1303,F1304,F130z,F13z0,F13z3,F13zz,F174.,F1740,F1741,F24y0,F24y2,Fyu20,Fyu21,Fyu22,Fyu29,Fyu2B,X003a,X003b,X003c,X003d,X003e,X003f,X003g,X003h,X003i,X003j,X003l,X003m,X003n,XaJgO,XaOfZ,XaQwf,XaQwg,XaZxQ,XaaRV,Xab7w,XabbC,XaeUM,XaeUN,d171.,dq...,dq1..,dq11.,dq12.,dq13.,dq14.,dq15.,dq16.,dq17.,dq18.,dq2.,dq21.,dq22.,dq2

				dq3K.,dq3L.,dq3M.,dq3N.,dq3O.,dq3P.,dq3Q.,dq3R.,dq3S.,dq3T.,dq3a.,dq3b.,dq3s.,dq3t.,dq3u.,dq3v.,dq3w.,dq3x.,dq3y.,dq3z.,dq4..,dq41.,dq42.,dq43.,dq44.,dq4z.,dq5..,dq51.,dq52.,dq53.,dq54.,dq55.,dq56.,dq57.,dq6..,dq61.,dq62.,dq63.,dq64.,dq65.,dq66.,dq67.,dq68.,dq69.,dq6A.,dq6w.,dq6x.,dq6y.,dq6z.,dq7..,dq8..,dq81.,dq82.,dq83.,dq84.,dq85.,dq86.,dq87.,dq88.,dq89.,dq8A.,dq9..,dq91.,dq92.,dq93.,dq94.,dq95.,dq96.,dq97.,dq98.,dq99.,dq9A.,dq9z.,dqA..,dqA1.,dqA2.,dqA3.,dqA4.,dqA5.,dqA6.,dqA7.,dqA8.,dqA9.,dqAA.,dqAB.,dqAC.,dqAD.,dqAa.,dqAb.,dqAc.,dqAd.,dqAe.,dqAf.,dqAg.,dqAh.,dqAi.,dqAj.,dqAk.,dqAl.,dqAm.,dqAn.,dqAo.,dqAp.,dqAq.,dqAr.,dqAs.,dqAt.,dqAu.,dqAv.,dqAw.,dqAx.,dqAy.,dqAz.,dqB..,dqB1.,dqB2.,dqB3.,dqB4.,dqB5.,dqB6.,dqC..,dqC1.,dqC2.,dqC3.,dqD..,dqD1.,dqD2.,dqE..,dqE1.,dqE2.,dqE3.,dqE4.,dqE5.,dqE6.,dqE7.,dqE8.,dqE9.,dqEA.,dqEB.,dqEo.,dqEp.,dqEq.,dqEr.,dqEs.,dqEt.,dqEu.,dqEv.,dqEw.,dqEx.,dqEy.,dqEz.,dqF..,dqF1.,dqFz.,dqG..,dqG1.,dqG2.,dqG3.,dqG4.,dqG5.,dqG6.,dqG7.,dqGt.,dqGu.,dqGv.,dqGw.,dqGx.,dqGy.,dqGz.	3.,dq24.,dq25.,dq26.,dq27.,dq28.,dq29.,dq2a.,dq2b.,dq2c.,dq3..,dq31.,dq32.,dq33.,dq34.,dq35.,dq36.,dq37.,dq38.,dq39.,dq3A.,dq3B.,dq3C.,dq3D.,dq3E.,dq3F.,dq3G.,dq3H.,dq3I,dq3J.,dq3K.,dq3L.,dq3M.,dq3N.,dq3O.,dq3P.,dq3Q.,dq3R.,dq3S.,dq3T.,dq3a.,dq3b.,dq3s.,dq3t.,dq3u.,dq3v.,dq3w.,dq3x.,dq3y.,dq3z.,dq4..,dq41.,dq42.,dq43.,dq44.,dq4z.,dq5..,dq51.,dq52.,dq53.,dq54.,dq55.,dq56.,dq57.,dq6..,dq61.,dq62.,dq63.,dq64.,dq65.,dq66.,dq67.,dq68.,dq69.,dq6A.,dq6w.,dq6x.,dq6y.,dq6z.,dq7..,dq8..,dq81.,dq82.,dq83.,dq84.,dq85.,dq86.,dq87.,dq88.,dq89.,dq8A.,dq9..,dq91.,dq92.,dq93.,dq94.,dq95.,dq96.,dq97.,dq98.,dq99.,dq9A.,dq9z.,dqA..,dqA1.,dqA2.,dqA3.,dqA4.,dqA5.,dqA6.,dqA7.,dqA8.,dqA9.,dqAA.,dqAB.,dqAC.,dqAD.,dqAa.,dqAb.,dqAc.,dqAd.,dqAe.,dqAf.,dqAg.,dqAh.,dqAi.,dqAj.,dqAk.,dqAl.,dqAm.,dqAn.,dqAo.,dqAp.,dqAq.,dqAr.,dqAs.,dqAt.,dqAu.,dqAv.,dqAw.,dqAx.,dqAy.,dqAz.,dqB..,dqB1.,dqB2.,dqB3.,dqB4.,dqB5.,dqB6.,dqC..,dqC1.,dqC2.,dqC3.,dqD..,dqD1.,dqD2.,dqE..,dqE1.,dqE2.,dqE3.,dqE4.,dqE5.,dqE6.,dqE7.,dqE8.,dqE9.,dqEA.,dqEB.,dqEo.,dqEp.,dqEq.,dqEr.,dqEs.,dqEt.,dqEu.,dqEv.,dqEw.,dqEx.,dqEy.,dqEz.,dqF..,dqF1.,dqFz.,dqG..,dqG1.,dqG2.,dqG3.,dqG4.,dqG5.,dqG6.,dqG7.,dqGt.,dqGu.,dqGv.,dqGw.,d

					qGx.,dqGy.,dqGz.,x002B,x002C,x01Bl,x01Bm,x01Bo,x01Bp,x01Br,x02Ln,x02MC,x03hv,x05qy

Supplementary Table 5. Definition of covariates

Covariates	Definition	UK Biobank field ID	Reference
Regular physical activity	Regular physical activity was defined as doing moderate activity for ≥150 minutes per week OR vigorous activity for ≥ 75 minutes per week OR equivalent combination.	864, 874, 884, 894, 904, 914, 10962, 10971	1
Healthy diet	Participants were considered to have a healthy diet if they consumed at least four of the following seven food groups:<br>(1) Fruits: ≥ 3 servings/day;<br>(2) Vegetables: ≥ 3 servings/day;<br>(3) Fish: ≥2 servings/week;<br>(4) Processed meats: ≤ 1 serving/week;<br>(5) Unprocessed red meats: ≤ 1.5 servings/week;<br>(6) Whole grains: ≥ 3servings/day;<br>(7) Refined grains: ≤1.5servings/day.	1289, 1299, 1309, 1319, 1329, 1339, 1349, 1369, 1379, 1389, 1438, 1448, 1458, 1468	1-3
Social isolation	The social isolation index was constructed from three questions: Filed ID 709, number of persons in household (1 point was given for living alone); Filed ID 709/10740, frequency of friend/family visits (1 point was given for answering about once a month, once every few months, never or almost never, or no friends or family outside household); Filed ID 6160, leisure/social activities (1 point was given for answering attending none of the listed leisure/social activities). Categories of social isolation were defined according to the total scores: (1) least isolated for score of 0; (2) moderately isolated for score of 1; and (3) most isolated for score of 2 or 3.	709, 1031, 6160, 10740	4
Mental health score	Data fields used to measure mental health score included mood swings, miserableness, irritability, sensitivity/hurt feelings, fed-up feelings, nervous feelings, worrier/anxious feelings, tense/highly strung, worry too long after embarrassment, suffer from nerves, loneliness/isolation, guilty feelings, and risk taking. The total mental health score were calculated by adding up participant's answers to the 13 UKB mental health questions, and higher numbers represent more mental health-related symptomatology.	1920, 1930, 1940, 1950, 1960, 1970, 1980, 1990, 2000, 2010, 2020, 2030, 2040	2
Family history of dementia	Family history was defined according to illnesses of father, mother, and siblings. Participants were considered to have a family history of dementia if any of the above family members reported Alzheimer's disease/dementia.	20107, 20110, 20111	

Supplementary Table 6. Comorbidities used to define Charlson comorbidity index[5, 6]

Comorbidities	ICD-10 code	ICD-9 code	Self-reported disease code
Myocardial infarction	I21.x, I22.x, I25.2, I25.5	410, 412	1075
Congestive heart failure	I11.0, I13.0, I13.2, I50	398.91, 402.01, 402.11, 402.91, 404.01, 404.03, 404.11, 404.13, 404.91, 404.93, 425.4–425.9, 428	1076
Peripheral vascular disease	I70, I71, I73.1, I73.8, I73.9, I77.1, I79.0, I792, K55.1, K55.8, K55.9, Z95.8, Z95.9	093.0, 437.3, 440, 441, 443.1–443.9, 47.1, 557.1, 557.9, V43.4	1067, 1087, 1379, 1380, 1492, 1591, 1592
Cerebrovascular disease	G45, G46, H34.0, I60-I69	362.34, 430–438	1081, 1082, 1083, 1086, 1491, 1583
Chronic pulmonary disease	I278, I279, J40-J47, J60-J67, J684, J701, J703	416.8, 416.9, 490–505, 506.4, 508.1, 508.8	1113, 1412, 1472, 1112, 1111, 1114, 1120
Connective tissue disease	M05, M06, M31.5, M32, M33, M34, M35.1, M353, M36.0	446.5, 710.0–710.4, 714.0–714.2, 714.8, 725	1373, 1377, 1381, 1383, 1384, 1464, 1480, 1481
Ulcer disease	K25-K28	531–534	1142, 1400, 1457
Mild liver disease	B18, K70.0-K70.3, K709, K71.3-K71.5, K71.7, K73, K74, K76.0, K76.2-K76.4, K76.8, K76.9, Z94.4	070.22, 070.23, 070.32, 070.33, 070.44, 070.54, 070.6, 070.9, 570, 571, 573.3, 573.4, 573.8, 573.9, V42.7	1155, 1156, 1157, 1158, 1506, 1578, 1579, 1580, 1581, 1582, 1604
Diabetes without complication	E10.2-E10.5, E10.7, E11.2-E11.5, E11.7, E12.2-E12.5, E12.7, E13.2-E13.5, E13.7, E14.2-E14.5, E14.7	250.0–250.3, 250.8, 250.9	1220, 1222, 1223
Hemiplegia	G041, G114, G801, G802, G81, G82, G830, G831-G834, G839	334.1, 342, 343, 344.0–344.6, 344.9	1252
Diabetes with chronic complication	E102-E105, E107, E112-E115, E117, E122-E125, E127, E132-E135, E137, E142-E145, E147	250.4–250.7	1276, 1468, 1607
Moderate or severe renal disease	I120, I131, N032-N037, N052-N057, N18, N19, N250, Z49.0-Z49.2, Z940, Z992	403.01, 403.11, 403.91, 404.02, 404.03, 404.12, 404.13, 404.92, 404.93, 582, 583.0–583.7, 585, 586, 588.0, V42.0, V45.1, V56	1192, 1193, 1194, 1515, 1519, 1520, 1608, 1609
Any tumor (including lymphoma and leukemia except for malignant neoplasm of skin)	C00-C26, C30-C34, C37-C41, C43, C45-C58, C60-C76, C81-C85, C88, C90-C97	140–172, 174–195.8, 200–208, 238.6	1001-1012, 1015-1048, 1050-1053, 1055, 1056, 1058-1068, 1072-1082, 1084, 1086-1088
Moderate or severe liver disease	I850, I859, I864, I982, K704, K711, K721, K729, K765-K767	456.0–456.2, 572.2–572.8	1141, 1158

Metastatic solid tumor	C77-C80	196–199	1070, 1071, 1085
HIV/AIDS	B20-B22, B24, Z21	042–044	1439
Head injury[7]	S02.0, S02.1, S02.8, S02.9, S04.02, S04.03, S04.04, S06, S071	800, 801, 803, 804, 850-854, 859	1266, 1626
Infection of CNS[8]	A06.6, A17.0, A17.1, A20.3, A32.1, A39.0, A50.4, A52.1-A52.3, A80, A81.1, A81.2, A83, A84, A85, A86, A87, A88, A89, B00.3, B00.4, B01.0, B01.1, B02.0, B02.1, B05.0, B05.1, B06.0, B15.0, B16.0, B16.2, B19.0, B22.0, B26.1, B26.2, B37.5, B38.4, B43.1, B45.1, B46.1, B50.0, B58.2, B60.2, B69.0, G00, G01, G02.0, G02.1, G02.8, G03, G04.1, G04.2, G05.0, G05.1, G05.2, G06, G07, I68.1, P35.0, P35.1	006.5, 013.0, 013.1, 027.0, 036.0, 090.4, 094, 045.0-045.2, 045.9, 046.2, 046.3, 062, 063, 048.0, 049.1, 064.0, 047, 049.0, 049.8, 049.9, 054.3, 052.0, 053.0, 055.0, 056.0, 070.0, 070.2, 072.1, 072.2, 112.8, 130.0, 136.2, 320, 332.9, 323.5, 323.8, 323.9, 324.0, 324.1, 324.9, 771.0,771.1	1244, 1245, 1246, 1247

Abbreviation: HIV/AIDS: Human immunodeficiency virus/ Acquired immune deficiency syndrome; CNS: Central nervous system.

References

1. Lourida I, Hannon E, Littlejohns TJ, et al. Association of lifestyle and genetic risk with incidence of dementia. *JAMA.* 2019;322(5):430-437.
2. Hepsomali P, Groeger JA. Diet, sleep, and mental health: insights from the UK Biobank study. *Nutrients.* 2021;13(8):2573.
3. Dobreva I, Marston L, Mukadam N. Which components of the Mediterranean diet are associated with dementia? A UK Biobank cohort study. *Geroscience.* 2022;44(5):2541-2554.
4. Smith RW, Barnes I, Green J, Reeves GK, Beral V, Floud S. Social isolation and risk of heart disease and stroke: analysis of two large UK prospective studies. *Lancet Public Health.* 2021;6(4):e232-e239.
5. Quan H, Sundararajan V, Halfon P, et al. Coding algorithms for defining comorbidities in ICD-9-CM and ICD-10 administrative data. *Med Care.* 2005;43(11):1130-1139.
6. Bannay A, Chaignot C, Blotiere PO, et al. The best use of the Charlson comorbidity index with electronic health care database to predict mortality. *Med Care.* 2016;54(2):188-194.
