# Supplementary material for: Prospective cohort study evaluating the association between influenza vaccination and neurodegenerative diseases
Source: NPJ Vaccines. 2024 Mar 2;9:51. doi: 10.1038/s41541-024-00841-z (PMC10908860; doi:10.1038/s41541-024-00841-z)
Supplement: Supplementary file 2 — REPORTING SUMMARY [file 41541_2024_841_MOESM2_ESM.pdf]

nature portfolio

Corresponding author(s): Siyan Zhan

Last updated by author(s): Jan 9, 2024

Reporting Summary

Nature Portfolio wishes to improve the reproducibility of the work that we publish. This form provides structure for consistency and transparency in reporting. For further information on Nature Portfolio policies, see our Editorial Policies and the Editorial Policy Checklist.

Statistics

For all statistical analyses, confirm that the following items are present in the figure legend, table legend, main text, or Methods section.

n/a	Confirmed	
☐	☒	The exact sample size (*n*) for each experimental group/condition, given as a discrete number and unit of measurement
☐	☒	A statement on whether measurements were taken from distinct samples or whether the same sample was measured repeatedly
☐	☒	The statistical test(s) used AND whether they are one- or two-sided<br>*Only common tests should be described solely by name; describe more complex techniques in the Methods section.*
☐	☒	A description of all covariates tested
☐	☒	A description of any assumptions or corrections, such as tests of normality and adjustment for multiple comparisons
☐	☒	A full description of the statistical parameters including central tendency (e.g. means) or other basic estimates (e.g. regression coefficient) AND variation (e.g. standard deviation) or associated estimates of uncertainty (e.g. confidence intervals)
☐	☒	For null hypothesis testing, the test statistic (e.g. *F*, *t*, *r*) with confidence intervals, effect sizes, degrees of freedom and *P* value noted<br>*Give P values as exact values whenever suitable.*
☒	☐	For Bayesian analysis, information on the choice of priors and Markov chain Monte Carlo settings
☒	☐	For hierarchical and complex designs, identification of the appropriate level for tests and full reporting of outcomes
☐	☒	Estimates of effect sizes (e.g. Cohen's *d*, Pearson's *r*), indicating how they were calculated

Our web collection on statistics for biologists contains articles on many of the points above.

Software and code

Policy information about availability of computer code

Data collection	Data were from the UK Biobank.
Data analysis	All statistical analyses were performed using SAS 9.4 (SAS Institute Inc., Cary, NC, USA) and R version 4.2.1.

For manuscripts utilizing custom algorithms or software that are central to the research but not yet described in published literature, software must be made available to editors and reviewers. We strongly encourage code deposition in a community repository (e.g. GitHub). See the Nature Portfolio guidelines for submitting code & software for further information.

Data

Policy information about availability of data

All manuscripts must include a data availability statement. This statement should provide the following information, where applicable:

- Accession codes, unique identifiers, or web links for publicly available datasets
- A description of any restrictions on data availability
- For clinical datasets or third party data, please ensure that the statement adheres to our policy

UK Biobank data are available online.

Research involving human participants, their data, or biological material

Policy information about studies with human participants or human data. See also policy information about sex, gender (identity/presentation), and sexual orientation and race, ethnicity and racism.

Reporting on sex and gender	We used the term sex in our manuscript. Both male and female participants were included. Information of participants' sex has been provided in the Method and Result sections.
Reporting on race, ethnicity, or other socially relevant groupings	Ethical characteristic was not used in this study. Education level was divided into University/college degree, A levels/AS levels or equivalent, O-levels/GCEs/CSEs or equivalent, NVQ/HND/HNC/other professional qualification, and Others. Townsend deprivation index (TDI) was classified according to quartiles. Average total household income was divided into Less than 18,000, 18,000 to 30,999, 31,000 to 51,999, and Greater than 52,000.
Population characteristics	Potential confounders were categorized as baseline and repeatedly measured covariates. All factors were collected at baseline through self-reported questionnaires, including sociodemographic characteristics (sex, age, education qualification, Townsend deprivation index (TDI), average household income, and region of assessment center), general health factors (self-reported health rating, family history of dementia/PD and body mass index [BMI]), mental health (mental health score), lifestyle (smoking and drinking status, diet, tea and coffee intake, physical activity, and social isolation). Further, the apolipoprotein E (ApoE) genotype was defined by the SNP rs429358 and rs7412. As ApoE ε4 is a well-recognized genetic risk factor for dementia, we divided the study population into ApoE ε4 carriers (+/+ or +/-) and noncarriers (−/−).
Recruitment	This study used data from the UKB, which recruited more than half a million participants of middle and old age across the United Kingdom in 2006-2010. All participants provided informed consent, completed touch-screen questionnaires and verbal interview, provided biological samples, and underwent physical examination. The UKB got initial ethical approval from its own Ethics Advisory Committee (https://www.ukbiobank.ac.uk/ethics/). We restricted our analyses to a subset of the entire cohort that could be linked to primary care data.
Ethics oversight	The UKB got initial ethical approval from its own Ethics Advisory Committee (https://www.ukbiobank.ac.uk/ethics/).

Note that full information on the approval of the study protocol must also be provided in the manuscript.

Field-specific reporting

Please select the one below that is the best fit for your research. If you are not sure, read the appropriate sections before making your selection.

☒ Life sciences ☐ Behavioural & social sciences ☐ Ecological, evolutionary & environmental sciences

For a reference copy of the document with all sections, see nature.com/documents/nr-reporting-summary-flat.pdf

Life sciences study design

All studies must disclose on these points even when the disclosure is negative.

Sample size	This study used data from the UKB, which recruited more than half a million participants of middle and old age across the United Kingdom in 2006-2010. We restricted our analyses to a subset of the entire cohort that could be linked to primary care data. A total of 70938 participants were included in the final analyses.
Data exclusions	We restricted our analyses to a subset of the entire cohort that could be linked to primary care data. We further excluded individuals aged < 60 years at baseline from the analysis as young people rarely develop dementia and Parkinson's disease (PD). We also excluded participants who received influenza vaccine or had a diagnosis of dementia or PD before the index date for identifying new receivers of FluVac and incident cases of dementia and PD.
Replication	All measures were described thoroughly to allow future replicability.
Randomization	N/A
Blinding	N/A

Reporting for specific materials, systems and methods

We require information from authors about some types of materials, experimental systems and methods used in many studies. Here, indicate whether each material, system or method listed is relevant to your study. If you are not sure if a list item applies to your research, read the appropriate section before selecting a response.

Materials & experimental systems

n/a	Involved in the study
☒	☐ Antibodies
☒	☐ Eukaryotic cell lines
☒	☐ Palaeontology and archaeology
☒	☐ Animals and other organisms
☐	☒ Clinical data
☒	☐ Dual use research of concern
☒	☐ Plants

Methods

n/a	Involved in the study
☒	☐ ChIP-seq
☒	☐ Flow cytometry
☒	☐ MRI-based neuroimaging

Clinical data

Policy information about clinical studies

All manuscripts should comply with the ICMJE guidelines for publication of clinical research and a completed CONSORT checklist must be included with all submissions.

Clinical trial registration	N/A
Study protocol	N/A
Data collection	This study used data from the UKB, which recruited more than half a million participants of middle and old age across the United Kingdom in 2006-2010. All participants provided informed consent, completed touch-screen questionnaires and verbal interview, provided biological samples, and underwent physical examination.
Outcomes	The primary outcomes were incident all-cause dementia and PD, which were ascertained through an algorithm combining self-reported medical conditions, linked data from hospital admissions, death registries, and primary care data. Incident cases were identified using Read v2, Read v3, and International Classification of Diseases 9th/10th (ICD-9/10) version codes, which are all given in the supplementary Table S3. This algorithm has been validated and has a positive predictive value of 82.5% for all-cause dementia and 91% for PD. We also defined three secondary outcomes including Alzheimer's disease (AD), vascular dementia (VD), and all other dementia (OD) except AD and VD.

Plants

Seed stocks	N/A
Novel plant genotypes	N/A
Authentication	N/A
